# Phylogeny of spiny frogs *Nanorana* (Anura: Dicroglossidae) supports a Tibetan origin of a Himalayan species group

**DOI:** 10.1002/ece3.5909

**Published:** 2019-12-05

**Authors:** Sylvia Hofmann, Chitra B. Baniya, Spartak N. Litvinchuk, Georg Miehe, Jia‐Tang Li, Joachim Schmidt

**Affiliations:** ^1^ Department of Conservation Biology UFZ – Helmholtz Centre for Environmental Research Leipzig Germany; ^2^ Central Department of Botany Tribhuvan University Kirtipur Nepal; ^3^ Institute of Cytology Russian Academy of Sciences St. Petersburg Russia; ^4^ Faculty of Geography Philipps University Marburg Marburg Germany; ^5^ Department of Herpetology Chengdu Institute of Biology Chinese Academy of Sciences Chengdu China; ^6^ Institute of Biosciences, General and Systematic Zoology University of Rostock Rostock Germany

**Keywords:** biodiversity, biogeography, Dicroglossinae, Himalaya, *Nanorana*, phylogeny

## Abstract

Recent advances in the understanding of the evolution of the Asian continent challenge the long‐held belief of a faunal immigration into the Himalaya. Spiny frogs of the genus *Nanorana* are a characteristic faunal group of the Himalaya–Tibet orogen (HTO). We examine the phylogeny of these frogs to explore alternative biogeographic scenarios for their origin in the Greater Himalaya, namely, immigration, South Tibetan origin, strict vicariance. We sequenced 150 *Nanorana* samples from 62 localities for three mitochondrial (1,524 bp) and three nuclear markers (2,043 bp) and complemented the data with sequence data available from GenBank. We reconstructed a gene tree, phylogenetic networks, and ancestral areas. Based on the nuDNA, we also generated a time‐calibrated species tree. The results revealed two major clades (*Nanorana* and *Quasipaa*), which originated in the Lower Miocene from eastern China and subsequently spread into the HTO (*Nanorana*). Five well‐supported subclades are found within *Nanorana*: from the East, Central, and Northwest Himalaya, the Tibetan Plateau, and the southeastern Plateau margin. The latter subclade represents the most basal group (subgenus *Chaparana*), the Plateau group (*Nanorana*) represents the sister clade to all species of the Greater Himalaya (*Paa*). We found no evidence for an east–west range expansion of *Paa* along the Himalaya, nor clear support for a strict vicariance model. Diversification in each of the three Himalayan subclades has probably occurred in distinct areas. Specimens from the NW Himalaya are placed basally relative to the highly diverse Central Himalayan group, while the lineage from the Tibetan Plateau is placed within a more terminal clade. Our data indicate a Tibetan origin of Himalayan *Nanorana* and support a previous hypothesis, which implies that a significant part of the Himalayan biodiversity results from primary diversification of the species groups in South Tibet before this part of the HTO was uplifted to its recent heights.

## Graphical abstract

Using multilocus nuclear and mitochondrial sequence data, phylogenetics, and molecular dating, we test different biogeographic scenarios of the evolution of the Himalayan spiny frogs (*Nanorana*). We found no evidence for a historical east–west range expansion of spiny frogs along the Himalaya, nor clear support for a strict vicariance model. In contrast, our data support a previous hypothesis, which implies that a significant part of the Himalayan biodiversity results from primary diversification of the species groups in South Tibet before this part of the orogeny was uplifted to its recent heights.

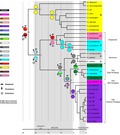

## 1 INTRODUCTION

For the past 45 ± 5 million years, the Earth has experienced one of the most dramatic continental collisions, when India collided with and was subducted beneath Asia (Gibbons, Zahirovic, Müller, Whittaker, & Yatheesh, 2015; Li et al., 2015; Lippert, van Hinsbergen, & Dupont‐Nivet, 2014; Molnar, Boos, & Battasti, 2010). The result, continuing today, has been the uplift of the most spectacular mountain ranges on Earth—the Himalaya, Karakoram, Pamir, and the Tibetan Highlands. Yet, although the collision time is widely accepted, various lines of geoscientific evidence have suggested—partly substantially—different elevational scenarios for the respective parts of the Himalaya–Tibet orogen (HTO) (Deng & Ding, 2015; Mulch & Chamberlain, 2006; Murphy et al., 1997; Tapponnier et al., 2001; C. Wang et al., 2008). Consequently, our understanding of the origin and historic biogeography of the terrestrial faunas inhabiting the HTO is far from conclusive, and has been hindered by a lack of and potential misinterpretation of data (Favre et al., 2015; Renner, 2016; Spicer, 2017; Su et al., 2019).

The key to disentangling the paleoclimatology and biogeography of the HTO lies particularly in the Himalaya (Molnar, 1986; Searle, 2013) for the following reasons: i) Most of the modern geological models differ with respect to the uplift history of this part of the mountain system (Schmidt, Opgenoorth, & Miehe, 2016, and refs. therein), ii) the uplift of the Greater Himalaya markedly influences the climate in the interior of High Asia and on a global scale (Boos & Kuang, 2010; Hodges, Hurtado, & Whipple, 2001; Sanwal et al., 2013), and iii) South Tibet may have been an important evolutionary center during the Cenozoic, which impacted the modern faunas of Central and East Asia (Schmidt, Opgenoorth, Holl, & Bastrop, 2012; Weigold, 2005).

So far, at least three different scenarios exist in the literature on the origin of the Himalayan wildlife: first, the long‐held belief of a faunal immigration scenario. Under this scenario, Himalayan taxa are assumed to have migrated along the Himalaya, that means, across preexisting barriers (deep valleys and high mountain ridges) (Martens, 2015). Many phylogenetic studies with a primary focus on the Greater Himalaya have been conducted in groups with higher dispersal potential, such as birds, butterflies, and plants with wind‐ or bird‐dispersed seeds (e.g., Deodati, Cesaroni, & Sbordoni, 2009; Favre et al., 2015; Mani, 1986; Martens, Tietze, & Päckert, 2011; Rajbhandary, Hughes, Phutthai, Thomas, & Shrestha, 2011; Xie, Ash, Linde, Cunningham, & Nicotra, 2014; Zhang, Kang, Zhong, & Sanderson, 2012). For the majority of these organisms, it has been reported that they originated through long‐distance dispersal from the mountains of China–Indochina along the southern slope of the Himalayan chain, associated with very little in situ speciation (Deodati et al., 2009; Johansson et al., 2007; Liu et al., 2013; Martens & Eck, 1995; Martens et al., 2011; Rajbhandary et al., 2011; Tabata, 2004; Xie et al., 2014; Zhang, Meng, Zhang, Vyacheslav, & Sanderson, 2014). For several Palearctic species groups, on the other hand, it has been shown that the influx of organisms came from the West along a climatically temperate corridor that enabled dispersal from Central Asia and the Pamiro‐Alai region into the Himalaya (Alcaide, Scordato, Price, & Irwin, 2014; Martens, 2015).

Second, recent studies of Asian forest‐dwelling beetles and anurans suggest a Tibetan‐origin scenario for at least some Himalayan faunal components (Hofmann et al., 2017; Schmidt et al., 2012). A similar explanation has been proposed for the evolution of highly isolated non‐wind‐dispersed Himalayan alpine plants (Miehe et al., 2015). This Tibetan‐origin scenario postulates that during the early phase of mountain uplift, South Tibet was an independent center of evolution that was well separated from other mountainous regions (Schmidt, 2006; Schmidt et al., 2012). According to this model, the final Plateau uplift, associated with the desiccation of the Tibetan highlands, “squeezed out” ancestral lineages that subsequently diversified by vicariance (Schmidt et al., 2012; Yin & Harrison, 2000). If so, adaptation to high altitudes and primary diversification of local species groups would have happened or at least been initiated in the high mountains of Paleo‐Tibet, potentially long before the final uplift of the Greater Himalaya. Colonization of the Greater Himalaya would have taken place in the course of its growth by ancestral species, originating in the immediately adjacent mountains to its north (i.e., at the southern edge of what is now Tibet). This area of origin, however, had been lost due to aridification, leading to faunal extinction or turnover there (Schmidt et al., 2012).

The third scenario is based on a previous study in *Nanorana* spiny frogs (Che et al., 2010). It implies a setting of explicit vicariance, driven by geographical isolation, climatic conditions, and an assumed low dispersal ability of spiny frogs. Accordingly, species formation among major lineages occurred as the species were “trapped” in the mountain mass and became separated when it uplifted (Che et al., 2010). However, in that study only three samples from the Himalaya were included, but none from the vast areas of the central and western parts of this mountain range.

Spiny frogs of the genus *Nanorana*, subfamily Dicroglossinae, are a characteristic species group of the HTO, living mostly in swift boulder‐strewn streams (Dubois, 1975). These frogs are found across the Himalayan arc from northern Pakistan and northern India, through Nepal, Sikkim, and Bhutan, to western China (Hengduan Shan), Myanmar, Thailand, Laos, northern Vietnam, and to montane southern China (Frost, 2019). The genus comprises 30 accepted species (Frost, 2019; Appendix 1) and is subdivided into three subgenera (*Nanorana* Günther, 1896, *Paa* Dubois, 1975, and *Chaparana* Bourret 1939; Frost, 2019). However, the phylogenetic and taxonomic relationships among spiny frogs, especially within the subgenera, but also within the subfamily, are still not completely resolved, as shown by taxonomic refinement during the last decade including the description of new species (Che et al., 2009; Frost, 2019; Huang et al., 2016; Jiang et al., 2005; Pyron & Wiens, 2011).

Herein, we explore whether the phylogeny of Himalayan *Nanorana* is better explained by an east‐to‐west immigration into the Himalaya, by a Tibetan‐origin scenario, or by a strict vicariance model. We generated a gene genealogy using mitochondrial and nuclear DNA fragments, reconstructed phylogenetic networks, and assessed the ancestral areas and the relative role of dispersal, vicariance, and extinction to examine the history of Himalayan spiny frogs. Based on the nuDNA, we also generated a time‐calibrated species tree.

In the case of an immigration scenario, the gene tree is expected to show nested phylogeographic distribution. The dating of the divergence between the lineages from the western part of the Greater Himalaya to a time younger than those at the eastern part would likewise argue for an immigration scenario, as would the occurrence of close relatives of Himalayan taxa in adjacent mountains. Gene flow among the regions at the southern slope of the Himalayan mountain chain from east to west or *vice versa* would result in a directional or intermingled nature of the haplotype patterns, due to (repeated) immigration into the Himalaya.

Under the Tibetan‐origin scenario, endemic Himalayan lineages are expected to show disjunct distribution with no close relationships to lineages occurring in adjacent parts of the mountain system, and which are of greater phylogenetic age. Himalayan lineages might be the same age as lineages from the eastern HTO (or might be even older than these), while lineages from the Tibetan Plateau should be closely related to any of the lineages from the HTO margin. Findings of shared haplotypes in adjacent drainage or mountain systems would provide evidence of dispersal in more recent times.

Under the strict vicariance scenario, dispersal of the lineages into the different parts of the HTO should have occurred more or less simultaneously during an early phase of the uplift. Lineages from the Tibetan Plateau are predicted to be at least as old as those occurring in the different parts of the Greater Himalaya. Consequently, similar to the Tibetan‐origin scenario, lineages are expected to be represented by deep phylogenetic branches and to correspond strongly to certain parts of the HTO. However, geographical ranges of sister lineages are not expected to overlap. We would expect no evidence for contemporary dispersal paralleling the Greater Himalaya, because barriers to dispersal in this part of the HTO were never as effective as today.

Our results may help to better understand the evolution and Cenozoic history of Himalayan biodiversity. They may also provide insights into how molecular phylogenies of poorly dispersing extant species groups can be integrated into advanced reconstructions of the highly complex geomorphological and paleoecological evolution of the HTO.

## 2 MATERIALS AND METHODS

### 2.1 Sampling and DNA extraction

**Figure 1:**
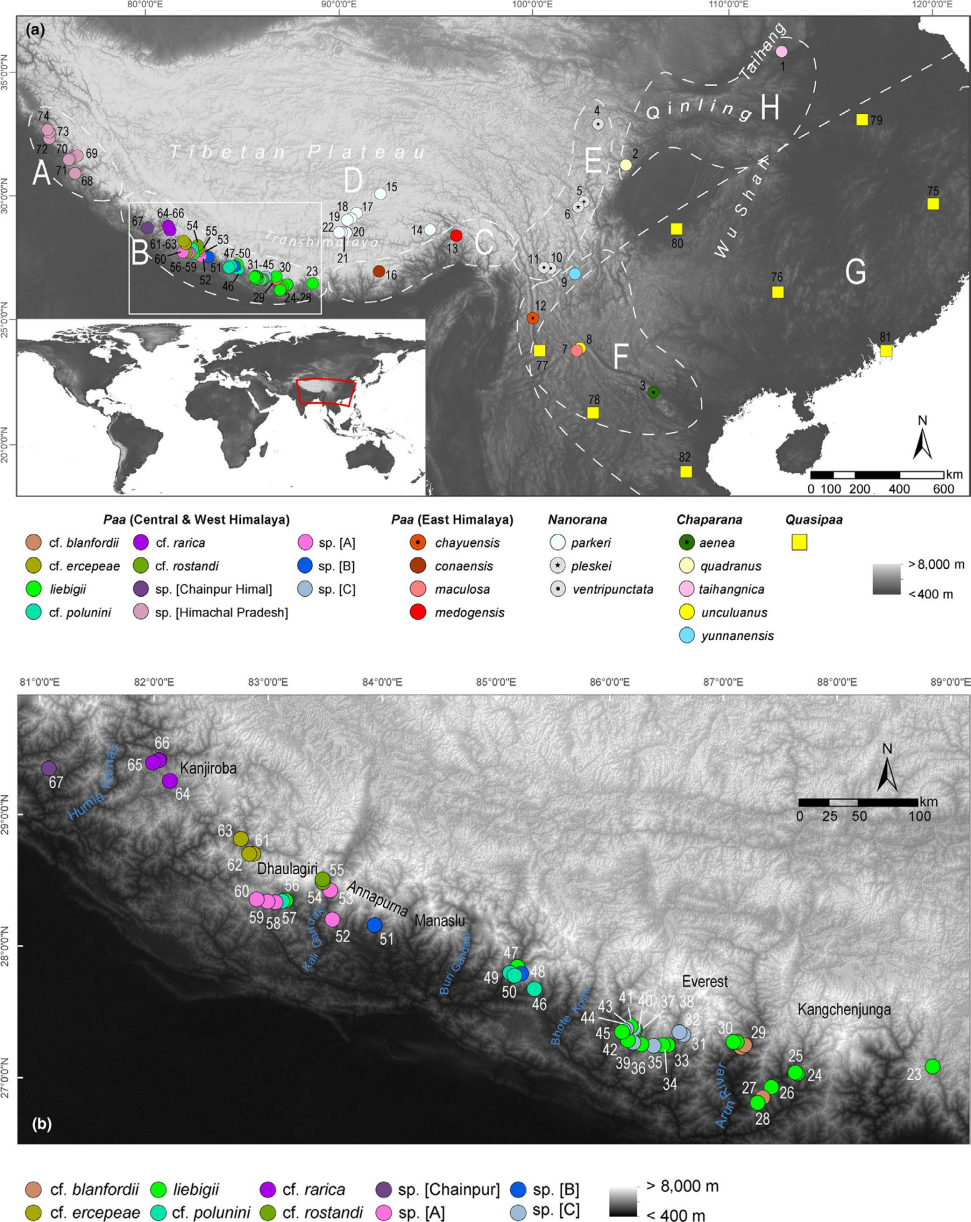
Map showing localities of sequences used in this study. (a) High Asia and adjacent regions, including the areas to which we assigned the samples of dicroglossid species (*A*‐*H*, dashed lined shapes) for the reconstruction of ancestral areas: *A* = NW Himalaya (Indus Himalaya), *B* = Central Himalaya (Sikkim to Uttar Pradesh = source area of the Ganges River), *C* = East Himalaya (source area of the Brahmaputra and its transverse valley); *D* = Transhimalaya and adjacent parts of the Tibetan Plateau; *E* = (sub)alpine parts of the eastern margin of the Tibetan Plateau; *F* = high‐montane regions of the southeastern margin of the Tibetan Plateau including the high mountains of northern Vietnam; *G* = subtropical and meridional eastern China; *H* = Sichuan Basin and mountains of Northeast China; (b) sampled area (zoomed rectangle as in Figure 1a), locality numbers refer to samples/ sequences listed in Appendix 2

A total of 150 individual samples of *Nanorana* spiny frogs covering major parts of the southern slope of the Himalaya chain were included in this study (Figure 1), coming from scientific collections (Chinese Academy of Science, CAS; Natural Museum of Erfurt, NME; Russian Academy of Science, RAS; Appendix 2). Samples were supplemented by NCBI sequence data from further *Nanorana* and outgroup species (Figure 1 and Appendix 2).

Genomic DNA was isolated from ethanol‐preserved tissues and noninvasive buccal swabs (Broquet, Berset‐Braendli, Emaresi, & Fumagalli, 2007) using the Qiagen DNeasy Tissue Kit or the PG‐AC4 PerformageneTM reagent package following the manufacturer's protocol. Partial sequences of the following three mitochondrial and three nuclear loci were amplified via polymerase chain reaction (PCR): *12S* rRNA (423 bp), *16S* rRNA (562 bp), *cytochrome oxidase subunit 1* (*co1*, 539 bp), *recombination activating protein 1 gene* (*rag1*, 1,210 bp), *rhodopsin* (*rhod*, 312 bp), and *tyrosinase* (*tyr*, 521 bp). Primers and conditions for PCR amplification are listed in Appendix 3.

### 2.2 Sequence alignment

Ribosomal RNA (rRNA) 12S and 16S sequences were aligned based on their secondary structures using RNAsalsa 0.8.1 (Stocsits, Letsch, Hertel, Misof, & Stadler, 2009) and the ribosomal structure model of *Bos taurus* downloaded from http://www.zfmk.de/en/research/research-centres-and-groups/rnasalsa. We generated a first structure‐based multiple sequence alignment using complete rRNA sequences of several *Nanorana* species (*N. yunnanensis*, *N. taihangnica*, *N. parkeri*, *N. pleskei*) and further dicroglossid species (*Fejervarya cancrivora*, *Hoplobatrachus rugulosus*, *Limnonectes fragilis*, *Quasipaa shini*) available from NCBI (Appendix 2). Remaining sequences were then aligned with this initial file using the MUSCLE algorithm (Edgar, 2004) in MEGA X (Kumar, Stecher, Li, Knyaz, & Tamura, 2018). To exclude ambiguously aligned sites, we used trimAL (Capella‐Gutierrez, Silla‐Martinez, & Gabaldon, 2009) under the *automated1* option, which implements a heuristic selection to decide the most appropriate mode depending on the alignment characteristics. The sequences of the protein‐coding genes were also aligned with MUSCLE using default settings in MEGA X. Alignment on the basis of nucleotides and amino acids produced similar results, since no ambiguities, such as deletions, insertions, or stop codons, were found.

### 2.3 Gene tree estimation

We inferred a maximum‐likelihood (ML) and a Bayesian inference (BI) tree based on the concatenated mtDNA + (unphased) nuDNA sequence data using RAxML version 8.2.10 (Stamatakis, 2014) and BEAST2 v. 2.5.1 (Bouckaert et al., 2014), respectively. We did not phase our nuclear data as many outgroup and ingroup taxa had only single representative individuals. The data set was partitioned a priori by gene fragments, and PartitionFinder 1.1.1 (Lanfear, Calcott, Ho, & Guindon, 2012) was applied to optimize partitions using linked branch lengths, the Bayesian information criterion (BIC), the “greedy” search algorithm, and the BEAST or RAxML option (Lanfear, Calcott, Kainer, Mayer, & Stamatakis, 2014). We ran RAxML with the GTRGAMMA model and 1,000 bootstrap replicates. BEAST2 analyses were based upon five independent runs with a chain length of 250 million each, thinning interval of 25,000, a lognormal relaxed clock model, a Yule tree prior, a random tree as starting tree, and the site models selected using bModelTest version 1.1.2 (Bouckaert & Drummond, 2017). Samples from these independent runs were compared, checked for convergence and stationary levels with Tracer v1.7.1 (Rambaut, Drummond, Xie, Baele, & Suchard, 2018), and combined after removing 10% of initial samples with LogCombiner v.2.5.1. We annotated the tree information with TreeAnnotator v.2.5.1 and visualized it with FigTree v.1.4.3.

### 2.4 Time‐calibrated species tree estimation

A time‐calibrated species tree was estimated using the uncorrelated relaxed clock method in *BEAST. Because mtDNA sequence data are expected to be systematically biased toward the calibration point and, thus, to overestimate divergence times (Zheng, Peng, Kuro‐o, & Zeng, 2011), we used only the unphased nuclear gene data for our dating analysis. This data set contained 182 taxa (of these, 32 were obtained from GenBank). We used the same run parameters as for the gene tree (i.e., five independent runs, 250 Mio generations, 25,000 sampling frequency, Yule speciation process, random starting tree, bModelTest on gene partitions), combined the runs after checking for convergence of modeled parameters, and annotated a maximum credibility clade tree using a burn‐in of the first 10% of the sampled trees.

Three carefully chosen fossil calibrations were selected to obtain divergence dates on the species tree and applied using an offset lognormal distribution: i) a minimum age of 33.9 Ma for the most recent common ancestor (MRCA) of Ranoidea based on the fossil *Thaumastosaurus gezei* (Rage & Roček, 2007), soft maximum 148.0 Ma (Feng et al., 2017) (95% CI: 34.5–148.0); ii) a minimum age of 25 for the MRCA of *Ptychadena* and *Phrynobatrachus* based on the earliest Ptychadenidae fossil (Blackburn, Roberts, & Stevens, 2015), soft maximum 148.0 Ma (Feng et al., 2017) (95% CI: 25.0–148.0); and iii) a minimum age of 15.97 Ma for the divergence of *Hyla cinereus* (North America) *versus.* *Hyla annectans* (Eurasia) based on the oldest fossils in Europe (Rage & Roček, 2003) (95% CI: 16.1–32.7; Roelants, Haas, & Bossuyt, 2011). Since anuran fossil records are notoriously rare and do not exist for dicroglossid frogs, we therefore relied on using previously published fossil‐calibrated divergence estimates to place a broad prior on the divergence of *Nanorana* and *Quasipaa* as further, internal calibration point. Based on Bossuyt et al. (Bossuyt, Brown, Hillis, Cannatella, & Milinkovitch, 2006), we used a calibration of 38.1 ± 9.4 Ma (28.7–47.5 Ma) for that node with a normal distribution prior. In all runs, we constrained Dicroglossinae to be monophyletic, as well as Microhylidae, *Nanorana*, and Natatanura, respectively; we also constrained *Sooglossus* to be the sister clade to Ranoidea since these relationships have been well established (Roelants et al., 2011; Wiens, Sukumaran, Pyron, & Brown, 2009; Zhang et al., 2013).

### 2.5 Haplotype network

We used SplitsTree4 v.14.8 (Huson & Bryant, 2006) and the NeighborNet algorithm based on the uncorrected p‐distances to construct a haplotype network of the concatenated mtDNA and (unphased) nuDNA sequence data set, respectively. To increase resolution, we reduced the two data sets to sequences that had less than 10% missing data (and less than 20% when analyzing mtDNA and nuDNA together).

### 2.6 Ancestral area reconstruction

To infer ancestral distributions and to elucidate the biogeographic history of Himalayan spiny frogs, we used two different approaches, namely Statistical Dispersal‐Extinction Cladogenesis (S‐DEC) and Statistical Dispersal‐Vicariance Analysis (S‐DIVA) implemented in RASP v.4.1beta (Yu, Harris, Blair, & He, 2015). Assignment of the samples to biogeographic units within the HTO proved to be difficult because current models of the spatiotemporal evolution of the HTO and of its biogeographic history are partly very different and no widely accepted model exists for the region. These differences arise particularly from biogeographic conclusions depending on whether they are based on distributional patterns of species groups with high dispersal ability (e.g., wind‐dispersed plants, butterflies, birds) or with very low dispersal ability (e.g., ground beetles; for an overview, see Martens, 2015). Because anurans disperse actively “on foot,” they are considered poor dispersers. They show, moreover, remarkable stasis in ecological niches, suggesting that dispersal will have been historically constrained between similar climatic conditions (Wiens, 2011). Due to the potentially restricted nature of their movements and dependence on relatively stable local environments, anuran species distributions should reflect a high level of paleoenvironmental history of a region. For the HTO, however, this history is still largely unknown (Renner, 2016). Therefore, we provisionally assigned the samples of dicroglossid species to certain parts of the HTO based on the species’ contemporary distribution and the delimitation of geological/geomorphological units as follows (see Figure 1): Greater Himalaya divided into the NW Himalaya (*A*; Indus Himalaya), Central Himalaya (*B*; Sikkim to Uttar Pradesh = source area of the Ganges River), East Himalaya (*C*; source area of the Brahmaputra and its transverse valley); Transhimalaya and adjacent parts of the Tibetan Plateau (*D*); (sub)alpine parts of the eastern margin of the Tibetan Plateau (*E*); high‐montane regions of the southeastern margin of the Tibetan Plateau including the high mountains of northern Vietnam (*F*); subtropical and meridional eastern China (*G*); Sichuan Basin and mountains of Northeast China (*H*). Units *E* and *F* differ mainly by their respective geological age, with the southeastern Plateau margin being the youngest part of the orogenic system with marked uplift just prior to 9–10 Ma (Clark et al., 2005; Schmidt et al., 2015). Species with wider distribution were assigned to the unit where most of the distributional area of the respective species is located. For example, *N. liebigi* has occasionally been reported from the westernmost part of the East Himalaya but is widely distributed in the Central Himalaya and is therefore assigned to the latter. *Nanorana quadranus* also occurs on the northeastern foothills of the Tibetan Plateau but is widely distributed along the Sichuan Basin (see Appendix 1) and consequently assigned to the latter. The northernmost records of *N. yunannensis* are located in Sichuan along the eastern Plateau margin; however, the distribution of this species is centered in the southeastern part of the HTO (unit *F*). Further, distributional areas of some species, for example, *Quasipaa spinosa* and *Q. verrucospinoa* (unit *G*), appear to overlap with species assigned to unit *F*, though these *G*‐assigned species occur at significantly lower elevations than those of the montane regions of the Plateau margin.

All ancestral state reconstructions were based on the post‐burn‐in trees of our BEAST2 analyses in order to explicitly incorporate phylogenetic uncertainty. To reduce computation time in RASP, we resampled trees from the posterior distribution of the five BEAST2 runs at lower frequency using LogCombiner v.2.5.1, resulting in 22,505 trees. The respective condensed tree was obtained with TreeAnnotator v.2.5.1. Because none of the taxa are distributed in more than two areas, we constrained the maximum range size by two. We excluded some unrealistic range combinations and allowed only contiguous composite ranges (except *AC*, as this combination corresponds to the Tibetan‐origin scenario). S‐DEC and S‐DIVA (two runs: allow reconstruction [“ar”] option disabled or enabled; “allow extinction” enabled) were performed with five threads; all other parameters were kept as default.

## 3 RESULTS

### 3.1 Sequence data characteristics

The final data sets (mtDNA + nuDNA; nuDNA) included sequences of 164 *Nanorana* samples, (14 derived from GenBank) and 18 outgroup species, including eight *Quasipaa* taxa, with a total of 3,567 bp (1,524 bp mtDNA, 2,043 bp nucDNA). Of these, 1,580 were variable (620 within the *Nanorana* group) and 1,170 being parsimony informative (498 in the *Nanorana* group).

### 3.2 Phylogenetic analyses

**Figure 2:**
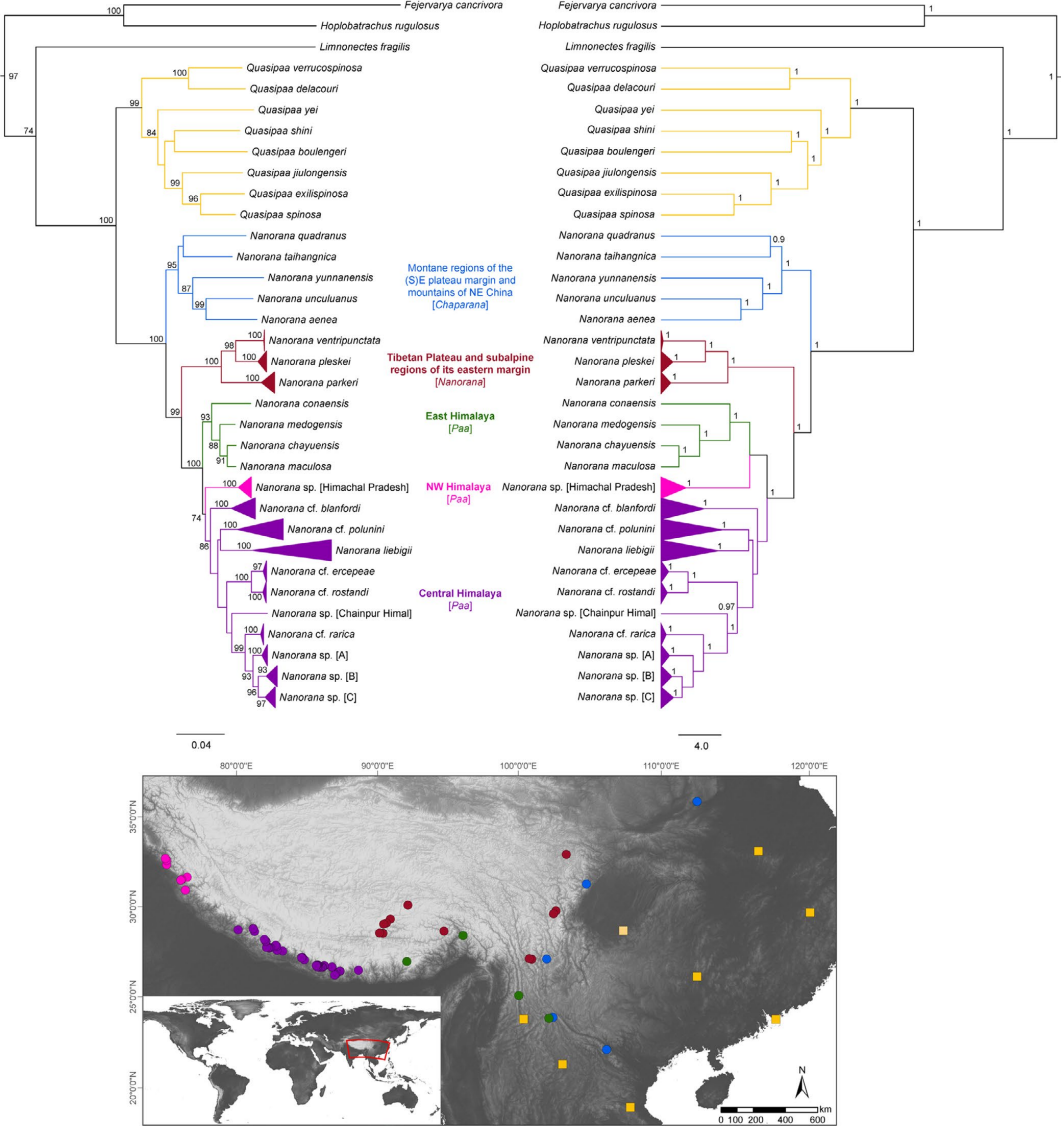
Maximum‐likelihood (left) and Bayesian inference tree (right) based on concatenated mtDNA and nuDNA sequence data. Numbers at branch nodes refer to bootstrap values > 70% (ML tree, 1,000 replicates) and or posterior probabilities ≥ 0.9 (Bayesian inference tree). The subgenus (*Chaparana*, *Nanorana*, or *Paa*) is also indicated at the respective clade. A map below the trees shows the localities of the samples within the phylogenetic clades; the color code corresponds to the color of the respective clade

The ML and BI gene trees (mtDNA + nuDNA) were well resolved and yielded almost identical tree topologies, except for the placement of the *Nanorana* lineage from the NW Himalaya, and for a few nodes of subclades from the Central Himalaya, which were weakly supported (Figure 2). All analyses supported two monophyletic clades, *Quasipaa* and *Nanorana*, with five subclades within *Nanorana*, namely from i) montane regions of the southeastern margin of the Tibetan Plateau and mountains of Northeast China (subgenus *Chaparana*); high‐montane regions of ii) the Northwest Himalaya (*Paa*; NWH), (iii) the East Himalaya (*Paa*; EH), and iv) the Central Himalaya (*Paa*; CH); and v) (sub)alpine regions of the Tibetan Plateau and its eastern margin (*Nanorana*). The Himalayan subgenus *Paa* forms the sister clade to the Plateau clade, which together constitute the sister clade of *Chaparana*. The results are largely consistent with a previous study (Che et al., 2010), except for two of the major clades, namely the NW and Central Himalaya clade, which shows further, significant substructure and which are highly relevant phylogenetic elements to unravel the historic biogeography of *Nanorana*. Haplotype network analysis also supports the strong divergences among the three major clades and the *Paa* lineages from the Himalaya (Appendix 4a, b); no directional or intermingled haplotype pattern is observed. Since the placement of the NW Himalaya clade is of particular interest in terms of the different biogeographic hypotheses (see Introduction), we tested the resulting topologies of major clades (best tree BEAST [m1: (NWH(CH))] and RAxML [m2: ((EH, NWH)CH)]) using a Bayes factor (BF) approach. The marginal likelihoods for the BF calculations were estimated under each model based on both the stepping stone (ss; Xie, Lewis, Fan, Kuo, & Chen, 2011) and path sampling (ps; Lartillot & Philippe, 2006) methods implemented in BEAST v. 1.10.4 (Suchard et al., 2018) using 250 million generations, a chain length of 1 million, and 100 path steps. Statistical support was then evaluated via 2lnBF using the ps/ss results as per Kaas & Raftery (Kass & Raftery, 1995). Optimal partitions and substitution models were assessed in PartitionFinder 1.1.1 (Lanfear et al., 2012) with branch lengths linked, a “greedy” search algorithm, the BIC, and the BEAST option (we run topology tests based on both, best scheme of gene partition and of genes + codon partition for protein‐coding genes). To reduce computation time, we included only data of dicroglossid frogs and excluded outgroups which had been used for the calibration analysis. The BF model selection clearly preferred model m1 based on both the stepping stone and path sampling method (Appendix 5). In fact, our split trees (Appendix 4) also showed a NW‐Central Himalaya sister group, especially based on nuclear genes.

The geographical distribution of the Himalayan lineages, which are strongly supported in the combined mtDNA and nuDNA data analyses, is presented in Appendix 6a‐d. These results show two apparently contradictory aspects. On the one hand, strong support is evident for local endemism of lineages to different valley systems within the Greater Himalayan mountain arc, even at intraspecific level. On the other hand, contemporary dispersal within, but also across, drainage boundaries is shown by some of the species investigated. For example, the phylogeographic pattern of the *liebigii* clade, which is monophyletic in all molecular data analyses and morphologically relatively easy to identify, even at the tadpole stage, shows significant sequence divergence of the populations along the Greater Himalaya. The deepest branches of this clade divide populations from different valleys and/or massifs (e.g., Arun and Kali Gandaki river valleys, Khumbu Himal, Kanchenjunga Himal; see Appendix 6a). Within these branches, lower but significant sequence divergence divides most of the populations that are geographically separated by deep gorges and south stretching mountain ridges. However, some populations extend across such barriers, suggesting contemporary dispersal events. Similar phylogeographic patterns are observed in *N.* cf. *blanfordii*, *N.* sp. [B], and *N.* cf. *polunini* (Appendix 6b and 6c).

### 3.3 Divergence times in spiny frogs and biogeographic reconstructions

**Figure 3:**
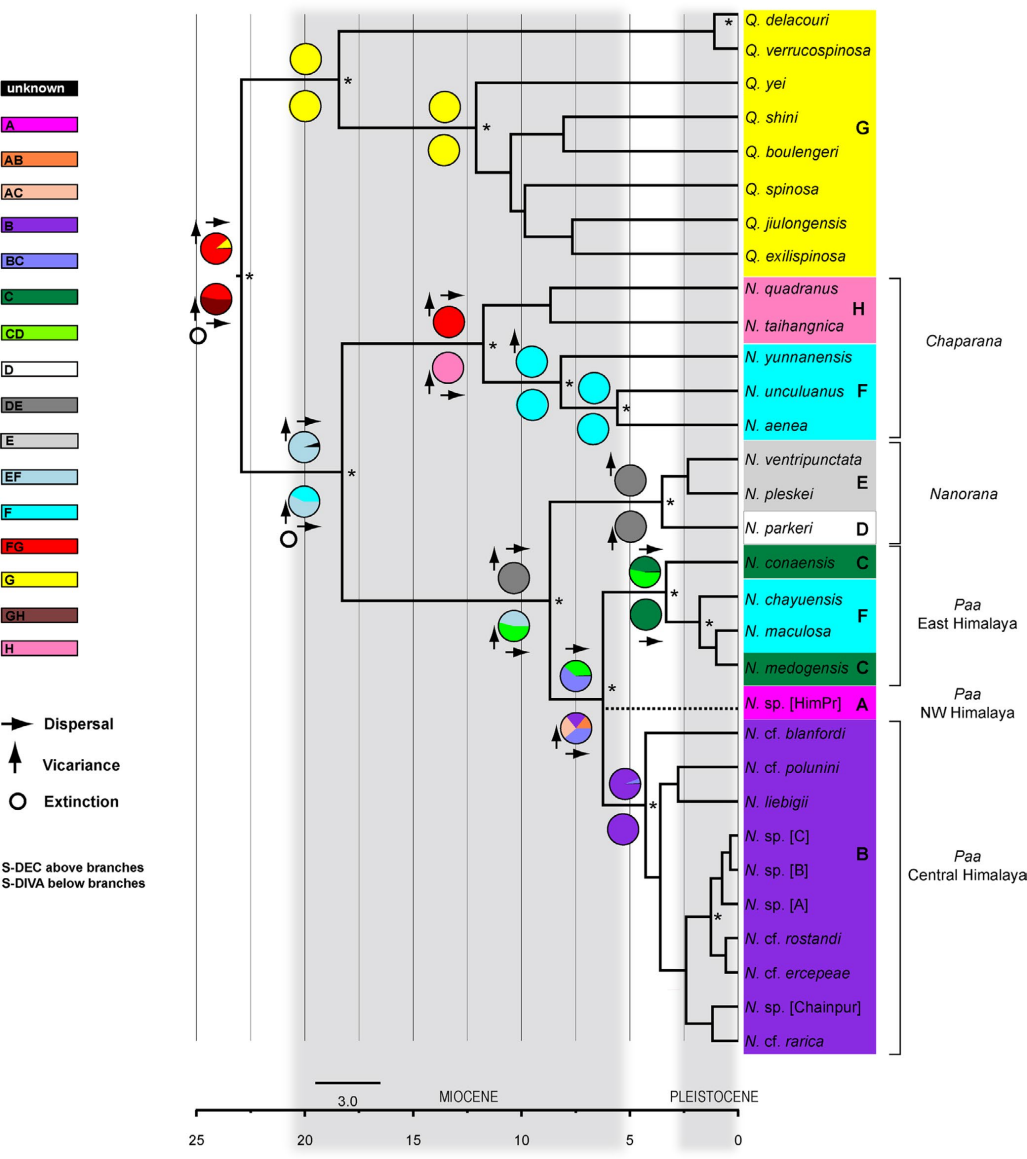
Time‐calibrated species tree of Asian spiny frogs using *BEAST and ancestral areas reconstructed with S‐DEC and S‐DIVA (above and below branches). Outgroups were excluded for readability. Bayesian posterior probability values ≥ 95% are indicated by an asterisk at the respective node. Codes in square brackets next to undescribed lineages (*N.* sp.) specify their region of origin (HimPr = Himachal Pradesh; Chainpur = Chainpur Himal) or simply a working label (A, B, C). *Nanorana* subgenera are indicated to the right of the clades. Pie charts at nodes show probabilities of alternative ancestral range for MRCAs obtained from S‐DEC and S‐DIVA, corresponding to the color key legend. Colored boxes around clades indicate their areas of distribution: *A* = NW Himalaya, *B* = Central Himalaya, *C* = East Himalaya; *D* = Transhimalaya and adjacent parts of the Tibetan Plateau; *E* = (sub)alpine parts of the eastern margin of the Tibetan Plateau; *F* = high‐montane regions of the southeastern margin of the Tibetan Plateau including the high mountains of northern Vietnam; *G* = subtropical and meridional eastern China; *H* = Sichuan Basin and mountains of Northeast China

Topology based on nuDNA was consistent with that based on the concatenated mtDNA and nuDNA sequence data (Figures 2 and 3). Dating analysis suggests an origin of the spiny frogs (*Quasipaa*, *Nanorana*) in the Lower Miocene (23 Ma, 95% HPD 11–35 Ma; Figure 3 and Appendix 7). This date is consistent with a transcriptome‐based study (Sun et al., 2018) and only slightly younger than the date reported by Che et al. (2010) who also recovered an Early Miocene or late Oligocene origin of these spiny frogs with an estimate of 22 Ma (12–34 Ma) and 27 Ma (19–36 Ma), respectively, but are approximately 15 million years younger than dates recovered by Bossuyt, and Roelants and colleagues (Bossuyt et al., 2006; Roelants, Jiang, & Bossuyt, 2004; Appendix 8). We found that both genera, *Nanorana* and *Quasipaa*, diversified in the Mid‐ to Late Miocene/Early Pliocene, although Che et al. (2010) date this radiation to the Early to Mid‐Miocene. Based on our data, first the subgenus *Chaparana* (distribution areas *F*, *H*; Figure 1a) split from *Nanorana* + *Paa* (18 Ma, 8–30 Ma), followed by the separation of *Nanorana* (Tibetan Plateau) from *Paa* (Greater Himalaya) around 9 Ma (3–16 Ma). The latter divergence estimate is considerably younger than that recovered by Che and colleagues (Che et al., 2010) but close to the date calculated by Sun et al. (2018) (13 Ma, 7–25) and Wiens et al. (2009) (10–12 Ma) (Figure 3 and Appendix 8).

The optimal reconstruction found by S‐DEC and S‐DIVA indicates that dispersal had more influence on lineage diversification of Asian spiny frogs than vicariance (S‐DEC: 15 dispersal events, 7 vicariance events, 4 extinctions; S‐DIVA: 13 dispersal events, 8 vicariance events, 4 extinctions; S‐DIVA results under “ar” option enabled or disabled were almost identical). The ancestor of *Hoplobatrachus*, *Fejervarya*, *Limnonectes, Nanorana,* and *Quasipaa* originated most probably from eastern China (area *G*, probability 1.0, S‐DEC and S‐DIVA; data not shown; for area references, see Figure 1a). From there, the ancestral lineages had spread westward and/or northward (area *FG*, probability 0.89, S‐DEC; *GH*, probability 0.53, S‐DIVA; Figure 3). Subsequent vicariance separated two lineages of ancestral spiny frogs, giving rise to *Nanorana* (area *EF*, probability 0.95, S‐DEC, and 0.57, S‐DIVA) and *Quasipaa* (area *G*, probability 1.0). *Quasipaa* further diversified across southern central and eastern China. Within *Nanorana*, dispersal across regions of the (south)eastern margin of the Tibetan Plateau (area *EF* up to *H*), potentially followed by vicariance, separated the lower montane *Chaparana* (areas *FG*, probability 1.0, S‐DEC, or *H*, probability 1.0, S‐DIVA) from the common ancestor of the high‐montane *Paa* and (sub)alpine *Nanorana* lineages (areas *DE*, probability 1.0, S‐DEC, or *CD*, probability 0.54, S‐DIVA). This contrasts with previous findings, which explained the separation of the subgenera explicitly by vicariant events (Che et al., 2010). Based on our results, ongoing colonization of regions north of today's Himalaya chain (Brahmaputra valley, Transhimalaya), and much later of the Tibetan Plateau, gave rise to the Himalayan *Paa* (area *BC*, probability 0.61, S‐DEC, and 0.39, S‐DIVA) and Tibetan *Nanorana* (area *DE*, probability 1.0). Further dispersal within the Himalayan group, followed by vicariance (S‐DIVA), separated the lineages that are found today in the Central Himalaya (area *B*, probability 0.94, S‐DEC, and 1.0, S‐DIVA) and those found across the Brahmaputra transverse valley (areas *CD*, probability 0.53 S‐DEC; *C*, probability 1.0, S‐DIVA). From the East Himalaya, lineages spread further to similar high‐montane regions along the eastern margin of the Plateau (area *F*). Noteworthy, for the NW Himalaya clade, which turned out to be most likely the basal sister clade to the Central Himalaya clade (see topology tests above), analyses also indicated an extinction event (S‐DEC) associated with the evolution of that far NW Himalaya clade and an origin of it in area *BC* (probability 1.0, S‐DEC) or *AC* (probability 1.0, S‐DIVA), data not shown.

## 4 DISCUSSION

### 4.1 Origin and evolutionary history of Himalayan spiny frogs

The commonly believed immigration scenario for faunal elements in the Himalaya assumes they originated via long‐distance dispersal, for example, from the mountains of China–Indochina along the Himalayan chain (see Hofmann et al., 2017; Schmidt et al., 2012 and references therein). Accordingly, as observed in birds, butterflies, and plants (e.g., Li et al., 2019; Manish & Pandit, 2018; Martens et al., 2011; Xie et al., 2014), all lineages of Himalayan spiny frogs recorded at present would have dispersed from east to west or would have appeared during the potential range expansion along the Greater Himalaya. Thus, they must have been able to cross the many north–south stretching mountain ranges and the epigenetic transverse valleys, by which the Himalaya has always been intersected and that might form effective barriers to dispersal of amphibians (Sánchez‐Montes, Wang, Ariño, & Martínez‐Solano, 2018; Zhou et al., 2017). Relating thereto, the phylogenetic placement of the clade from the NW Himalaya is of particular interest. Based on the immigration model, it can be expected that the NW Himalaya clade represents a terminal lineage of the highly diverse Central Himalaya clade. However, in all our analyses, the NW Himalaya clade was placed basally relative to the Central Himalaya clade (model m1) or as sister clade to the East Himalaya clade (model m2), though m1 had a significant higher likelihood than m2 based on the topology tests (Appendix 5). This not only indicates the presence of ancestral lineages in the NW Himalaya but also provides a strong argument against dispersal of spiny frogs paralleling the Greater Himalayan mountain arc.

The results of our analyses demonstrate that the ancestral Asian spiny frogs descended, during the Lower Miocene, from a tropical ancestor in eastern China that gave rise to the genera *Quasipaa* and *Nanorana*, with *Nanorana* comprising three major clades (subgenera), namely the lower montane *Chaparana*, the (high‐)montane *Paa*, and the (sub)alpine, nominal subgenus *Nanorana*. Up to this point, our results confirm previous findings (Che et al., 2010). However, we recovered a highly supported distinct Central Himalayan *Paa* clade, which rapidly diversified during the Pliocene and Pleistocene. It includes the high‐montane species *Nanorana liebigii*, *N*. cf. *blanfordii*, *N*. cf. *ercepeae*, *N*. cf. *polunini*, *N.* cf. *rarica*, *N*. cf. *rostandi*, and several undescribed lineages from the southern slopes of the Himalaya. This clade shows strong phylogeographic structure and constitutes—most parsimoniously together with the NW Himalaya clade—the sister clade to high‐montane *Paa* species occurring in the East Himalaya and the adjacent high‐montane regions of the southeastern margin of the Tibetan Plateau. According to our dating results, the MRCA of these sister clades (*Paa*) and the Plateau clade (*Nanorana*) occurred in the Upper Miocene, *ca.* 9 Ma. The MRCA of *Chaparana* and *Nanorana *+ *Paa* occurred almost ten million years earlier, during the Lower Miocene (*ca*. 18 Ma). A similar age for that split has been found in other studies (Che et al., 2010; Chen et al., 2017; Wiens et al., 2009). Between these two nodes, the ancestral lineages of *Nanorana* + *Paa* apparently did not diversify for several million years before major diversification occurred. There are two alternative explanations for such a pattern. Either these ancestral lineages persisted in their area of origin, which had remained stable over a long period until it facilitated new habitats (or niches), or this ancestral area successively disappeared in the course of the stepwise uplift and drying of Tibet led to extensive species extinction and range shifts into new suitable areas. The first scenario is unlikely because mountain building is accompanied by both gradual and abrupt environmental changes, which ultimately drive biodiversity dynamics (Huang, Meijers, Exres, Mulch, & Fritz, 2019). The latter explanation corresponds to the theory of a Tibetan origin of Himalayan spiny frogs. The genetic data presented here strongly suggest that the Himalayan spiny frogs originated to the north of the Greater Himalaya, that is, the southern margin of the HTO during an early phase of uplift of the orogenic system, probably in regions of the Transhimalaya (area *DE*/*CD*, S‐DEC and S‐DIVA; Figures 1a and 3). Our dating places the immigration of the *Nanorana*/*Paa* ancestor into the HTO between the Lower and the Mid‐Miocene. A remarkably similar time was reported for the ancestor of the ground beetle *Ethira* clade, which shows a similar phylogeography to that of the spiny frogs and for which a Tibetan‐origin scenario has been demonstrated (Schmidt et al., 2012). Our data indicate a scenario under which the ancestor of Himalayan spiny frogs had adapted to the high altitude environment in South Tibet, most likely during the Mid‐Miocene, prior to the final uplift of the Greater Himalaya (Wang, Shi, & Zhou, 1982; Wang et al., 2008). Due to the rising Himalayan mountain belt and the associated continuous aridization of southern Tibet, many ancestral lineages might have successively been lost to extinction or been forced to track the displaced suitable environment along the transverse valleys of the Himalaya, such as the Arun, Brahmaputra, Kali Gandaki, or the Indus catchment (see Figure 1b and Appendix 1 for geographical reference), which otherwise formed an effective barrier against the north–south dispersal of these species (Schmidt et al., 2012). Shifting ranges in response to substantial climatic changes is a common phenomenon that defines phylogeography especially in temperate taxa and mountain biota (Giezendanner, Bertuzzo, Pasetto, Guisan, & Rinaldo, 2019; Hoorn, Perrigo, & Antonelli, 2018). The strong restriction of the modern geographical distribution of subclades in the Greater Himalaya and their deep phylogenetic splits (Appendix 6a‐d) can be explained by such ancient migration corridors and the severely limited gene flow across them. The long branching pattern between the splits of *Chaparana *versus*. Nanorana/Paa* and *Nanorana *versus*. Paa* additionally supports the Tibetan‐origin scenario by indicating a stepwise eradication process that may have taken place in the inner parts of the HTO (Figure 3).

Considering this scenario, it becomes evident why the NW Himalaya clade of spiny frogs is by far the most separated lineage geographically. Apparently, this lineage evolved on the western macro‐slope of southern Paleo‐Tibet, which is drained by the Indus catchment. Like all of the other main drainage systems of the southern HTO, the Indus River originates from the central part of the orogeny (see Appendix 1). With the progressive uplift and associated drying of Tibet, the ancestors of the modern Himalayan *Paa* species were forced to track the displaced habitats along slopes of the Tibetan Himalaya and the Transhimalaya paralleling the upper Indus valley more than 500 km to the northwest until reaching the transverse Indus valley in the NW Himalaya. A similar pattern was observed in the *Ethira* clade of *Pterostichus* ground beetles (Schmidt et al., 2012). The Kashmir Himalaya may be considered an “exile area,” meaning it is the distributional area of the *Paa* descendants which originated from the western slope of Paleo‐South Tibet. Similarly, from the eastern macroslope of the HTO, ancestral species followed the main river gorges into the southeastern Plateau margin (see modern distribution of *N. chayuensis, N. maculosa* in Appendix 1 and Figure 1a; for evolution of the great river systems see Brookfield, 1998; Lang & Huntington, 2014).

Further support for a South Tibetan‐origin scenario is provided by the phylogeographic pattern of the Tibetan Plateau frog (*N. parkeri*), which is the only known *Nanorana* taxon that occurs in the alpine zone while all other members of this genus are high‐montane to subalpine taxa. This points to a stepwise high altitude adaptation. The relatively recent divergence time between the alpine *N. parkeri* and the subalpine *N*. *pleskei*/*N. ventripunctata* from the eastern Plateau margin (< 5 Mya) fits the concept of a final uplift of the Tibetan Plateau rather late in the Neogene (Su et al., 2019). An almost identical biogeographic history has been demonstrated in *Scutiger boulongeri*, the Tibetan alpine toad (Hofmann et al., 2017).

In contrast to the Tibetan‐origin scenario, the strict vicariance model suggests that spiny frogs colonized the uplifting HTO and were then “trapped” in their habitats when the mountains evolved (Che et al., 2010). Considering this scenario, we must assume that, for example, the spiny frog lineage that occurs on the central Tibetan Plateau (*N. parkeri*) reflects its ancestral spatial distribution and that the uplifting Tibetan Plateau triggered further diversification within that lineage. Since modern geological models consistently suggest that at least the southern central part of the Tibetan Plateau had reached significant heights before the Greater Himalaya was uplifted (Deng & Ding, 2015; Mulch & Chamberlain, 2006; Wang et al., 2008), the Plateau lineage is expected to branch basally relative to the other clades occurring in the Greater Himalaya. However, *N. parkeri* clusters together with species from the eastern Plateau margin, representing a relatively young, terminal branch as discussed above (Figure 2). For the current distribution of spiny frogs in the Greater Himalaya, two alternative explanations emerge under the vicariance model. On the one hand, the ancestral *Paa* lineages may have colonized the different parts of the Greater Himalaya before this mountain arc was uplifted. Consequently, these lineages must have been elements of a tropical fauna, while the *Nanorana* lineage of the Tibetan Plateau must have been already adapted to high altitudes. This implies that the ancestral species were able to adapt to new conditions under a dramatically changing environment in their current locations. Given the existence of subtropical vegetation (cloud forests) in the Tibetan Himalaya north of the Central Himalaya during the Miocene and even during the Late Pliocene, as demonstrated by plant fossil data (Su et al., 2019; Xu, 1981, 1982), this environment must have been rapidly transformed from subtropical montane to arid alpine habitats. However, since most amphibian populations can only accommodate very gradual environmental changes, their phenotypic plasticity and genetic variance might be insufficient to generate the phenotypic changes necessary to cope with such massively transformed conditions (Urban, Richardson, & Freidenfelds, 2014).

On the other hand, like the immigration scenario, the ancestral *Paa* lineages may have dispersed parallel to the growing Greater Himalayan mountain arc—a concept that is not supported by our data as we have discussed above.

A central argument of the strict vicariance scenario is the geographical and ecological separation of *Nanorana* lineages induced by the uplift of the Himalayan and Tibetan region. This argument, however, is compatible neither with a distribution that stretches over several mountains and deep valleys (Appendix 6a‐d), nor with the overlapping altitudinal ranges (Hu, Xie, Li, & Jiang, 2011) and sympatry of some spiny frog species, e.g., *Chaparana yunnanensis*, *C. unculuanus*, and *Paa maculosa*; Appendix 1). Given that barriers to dispersal were never as high as in the present HTO, the modern distributional patterns raise questions about a strict vicariance scenario.

### 4.2 Conclusions

Phylogeography of the HTO encompasses approximately 50 million years of evolution and one of the most complex mountain systems on the planet. Given the resulting biodiversity, generalizations are difficult, but some patterns, particularly those that are found congruent among different taxonomic groups, are especially informative. Our results show strikingly similar phylogeographic patterns as observed in other Himalayan faunal elements that are assumed to have originated in South Tibet, for example, forest‐dwelling *Pterostichus* ground beetles (Schmidt et al., 2012) and *Scutiger* lazy toads (Hofmann et al., 2017). These patterns are indicated by i) the presence of several distinct lineages of a monophyletic clade each occurring in restricted areas along the Greater Himalaya, ii) the presence of at least one ancient lineage endemic to the central portion of the Himalaya, and iii) lineages which are geographically adjacent are not necessarily closely related. Indeed, genetic separation, diversity, and geographical distribution of Himalayan spiny frogs seem to be best explained by a South Tibetan origin rather than by the alternative immigration or strict vicariance scenario. A Tibetan origin of high‐montane faunal elements of the Himalaya is coherent with isotope records as well as with fossil evidence of Late Paleogene and Miocene subtropical to temperate vegetation north of the Greater Himalaya (Su et al., 2019; Wang, Deng, & Biasatti, 2006; Xu et al., 2012), suggesting the existence of cloud forests in Paleo‐Tibet.

Although our molecular data markedly support a Tibetan origin for Himalayan spiny frogs, some ambiguities remain, for example, in the phylogenetic placement of some Himalayan lineages. Denser sampling in additional mountain systems of the Greater Himalaya and the use of a more extensive set of nuclear markers, for example, through the use of next‐ or third‐generation sequencing, will be required to improve the resolution for relationships of Himalayan spiny frogs and allow the tracking of the true pattern of their cladogenesis. Potentially, this would also reveal numerous additional, undescribed lineages.

We hope that this work will encourage further studies aiming to analyze the biogeographic history in a range of additional taxonomic groups in the Himalaya, to assess the evolutionary significance of the HTO and to understand modern distributional patterns against the background of existing geological scenarios.

## CONFLICT OF INTEREST

None declared.

## AUTHOR CONTRIBUTION

S.H. and J.S. performed conceptualization. S.H., C.B.B., J.S., S.N.L., and J.‐T.L. performed methodology. S.H. performed formal analysis. S.H., J.S., and C.B.B. interpreted the data. S.H., C.B.B., J.S., S.N.L., and J.‐T.L. contributed to resources. S.H. contributed to writing—original draft preparation. All authors contributed to writing—review and editing. S.H. performed visualization. S.H. involved in the project administration. S.H., J.S., and G.M. involved in the funding acquisition.

## Data Availability

All sequences were uploaded to GenBank; details on individual samples and accession numbers are available in Appendix 2.
