# Supplementary figures and images for: Phylogeny of spiny frogs Nanorana (Anura: Dicroglossidae) supports a Tibetan origin of a Himalayan species group
Source: Ecol Evol. 2019 Dec 5;9(24):14498–511. doi: 10.1002/ece3.5909 (PMC6953589; doi:10.1002/ece3.5909)

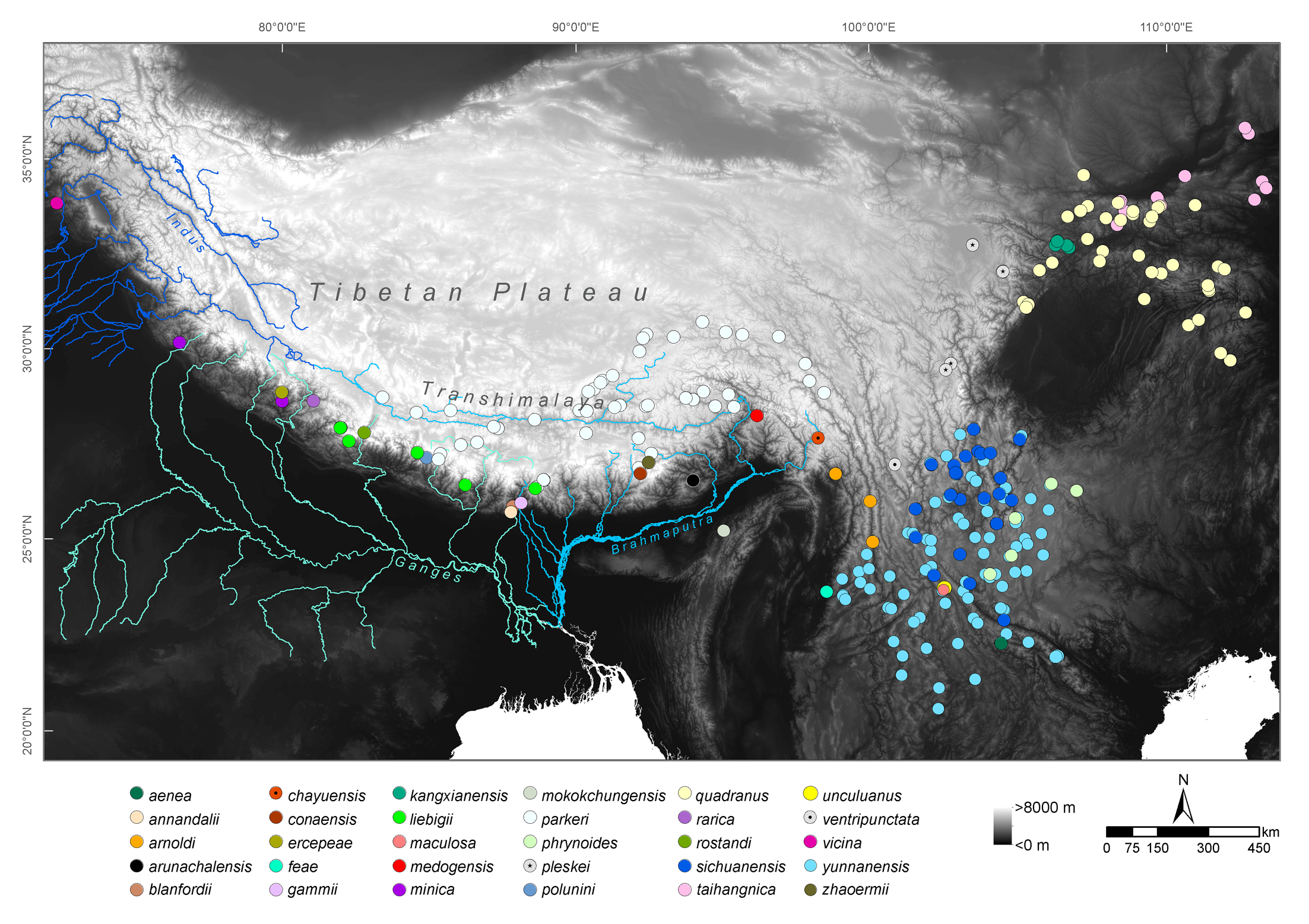

Supplement: Supplementary file 1 [file ECE3-9-14498-s001.tif]

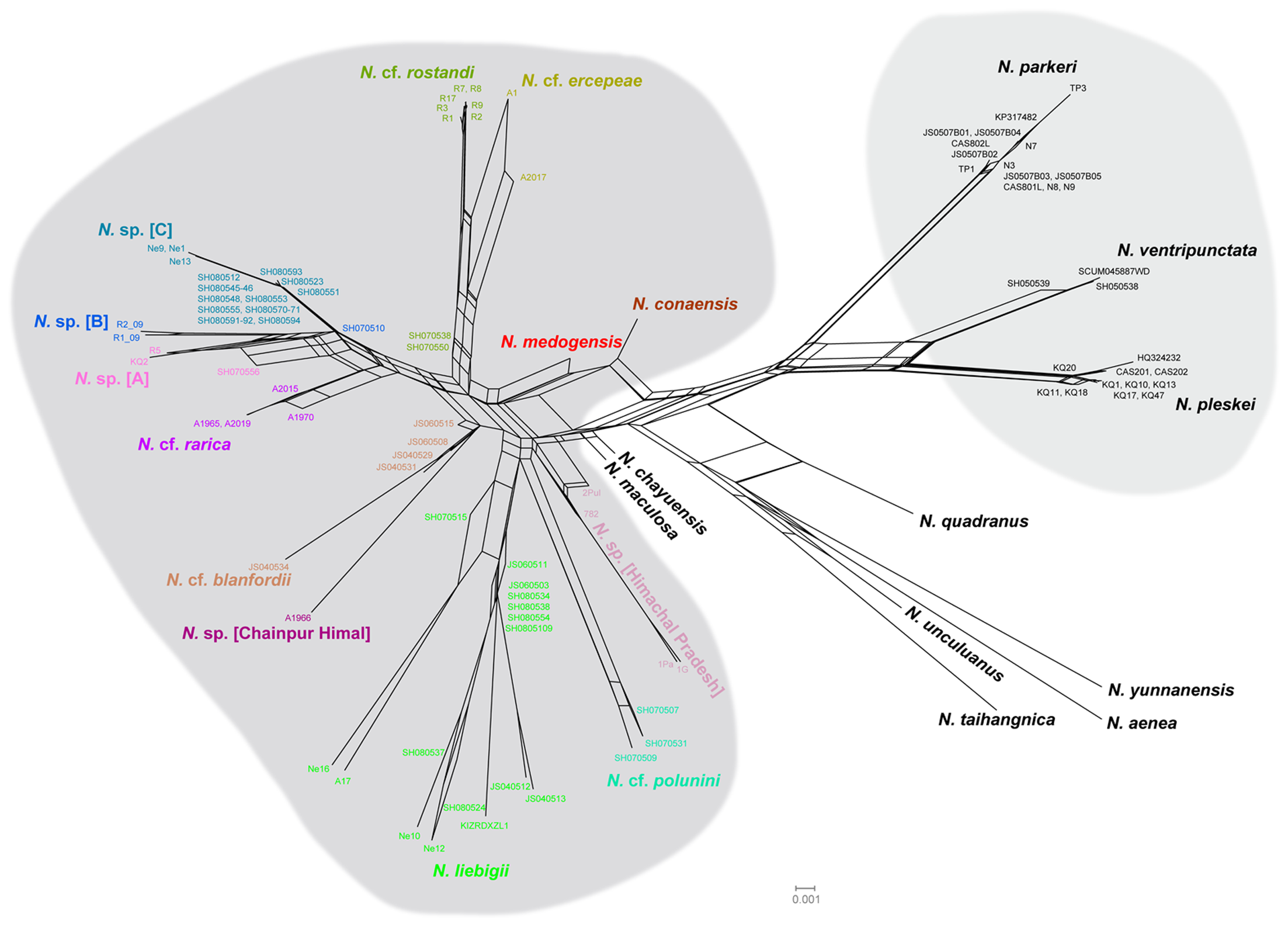

Supplement: Supplementary file 4 [file ECE3-9-14498-s004.tif]

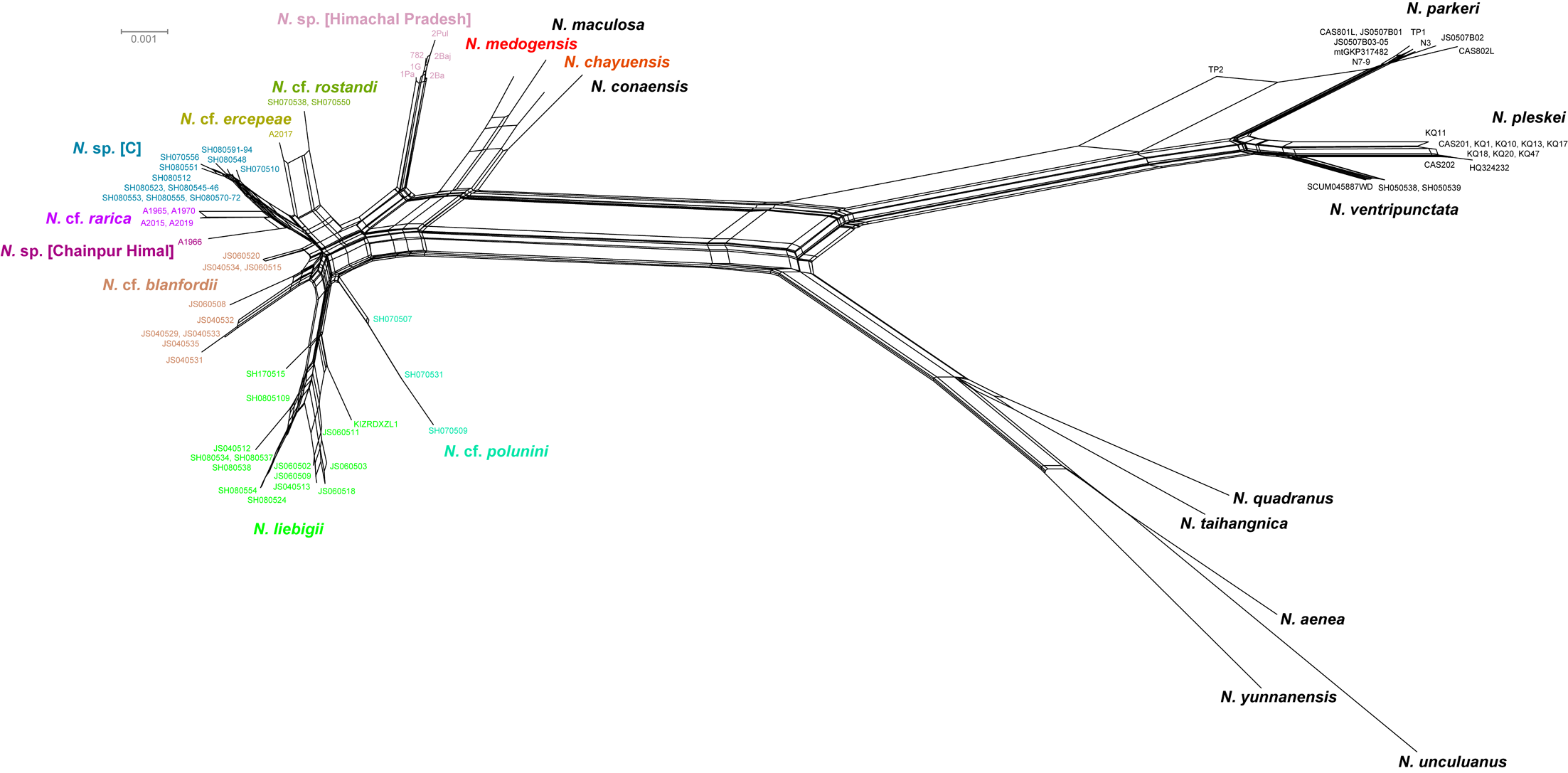

Supplement: Supplementary file 5 [file ECE3-9-14498-s005.tif]

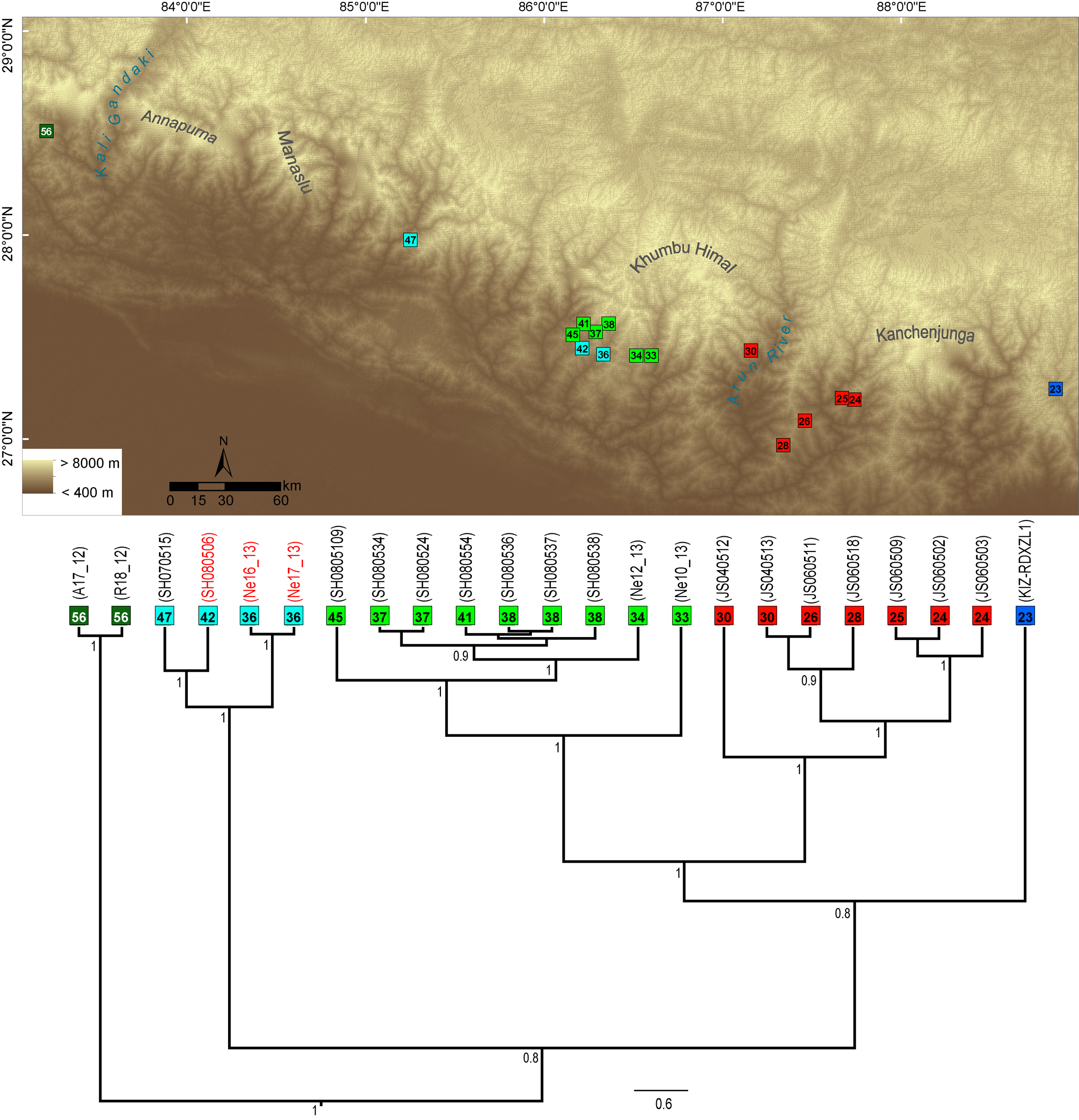

Supplement: Supplementary file 7 [file ECE3-9-14498-s007.tif]

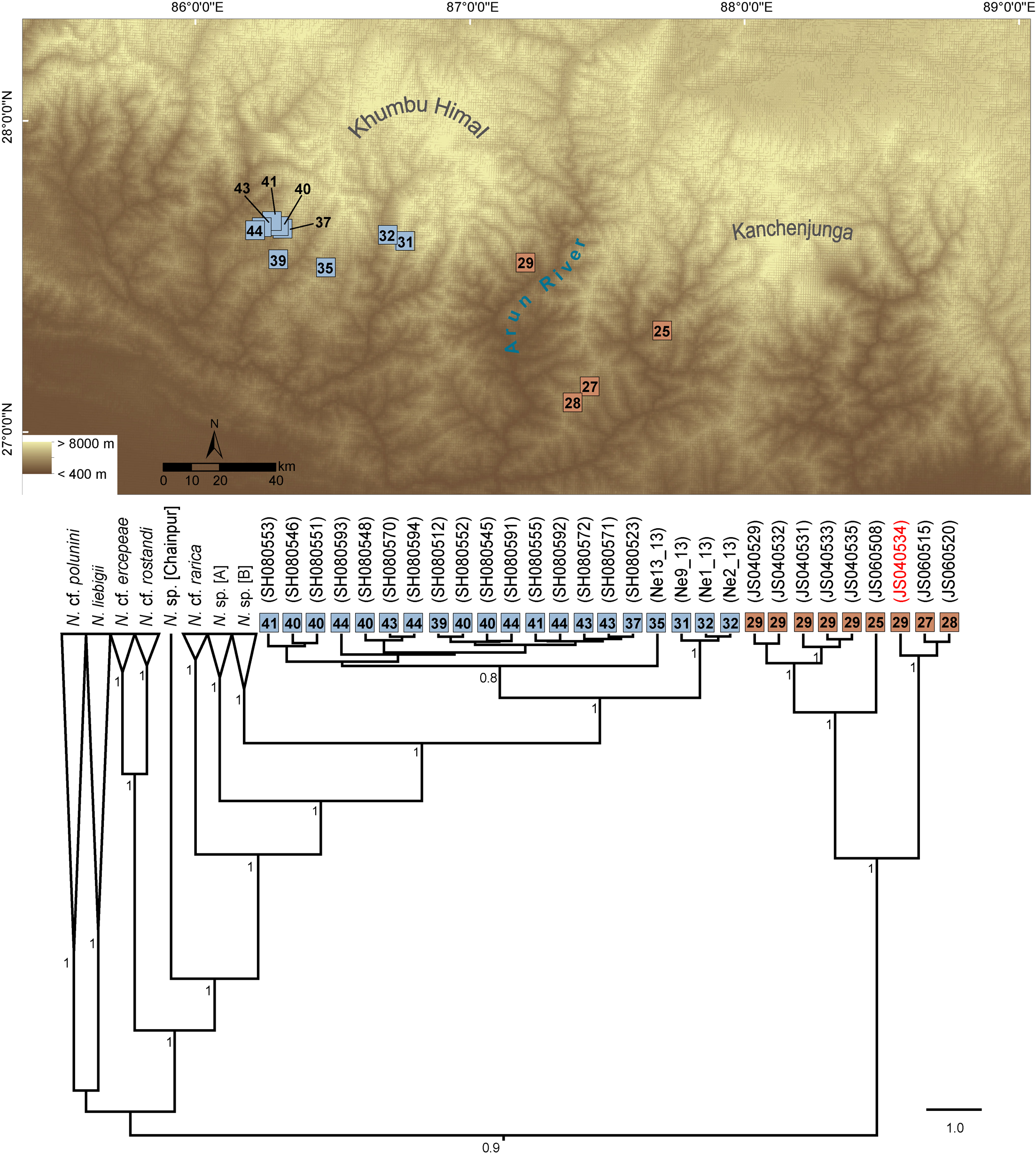

Supplement: Supplementary file 8 [file ECE3-9-14498-s008.tif]

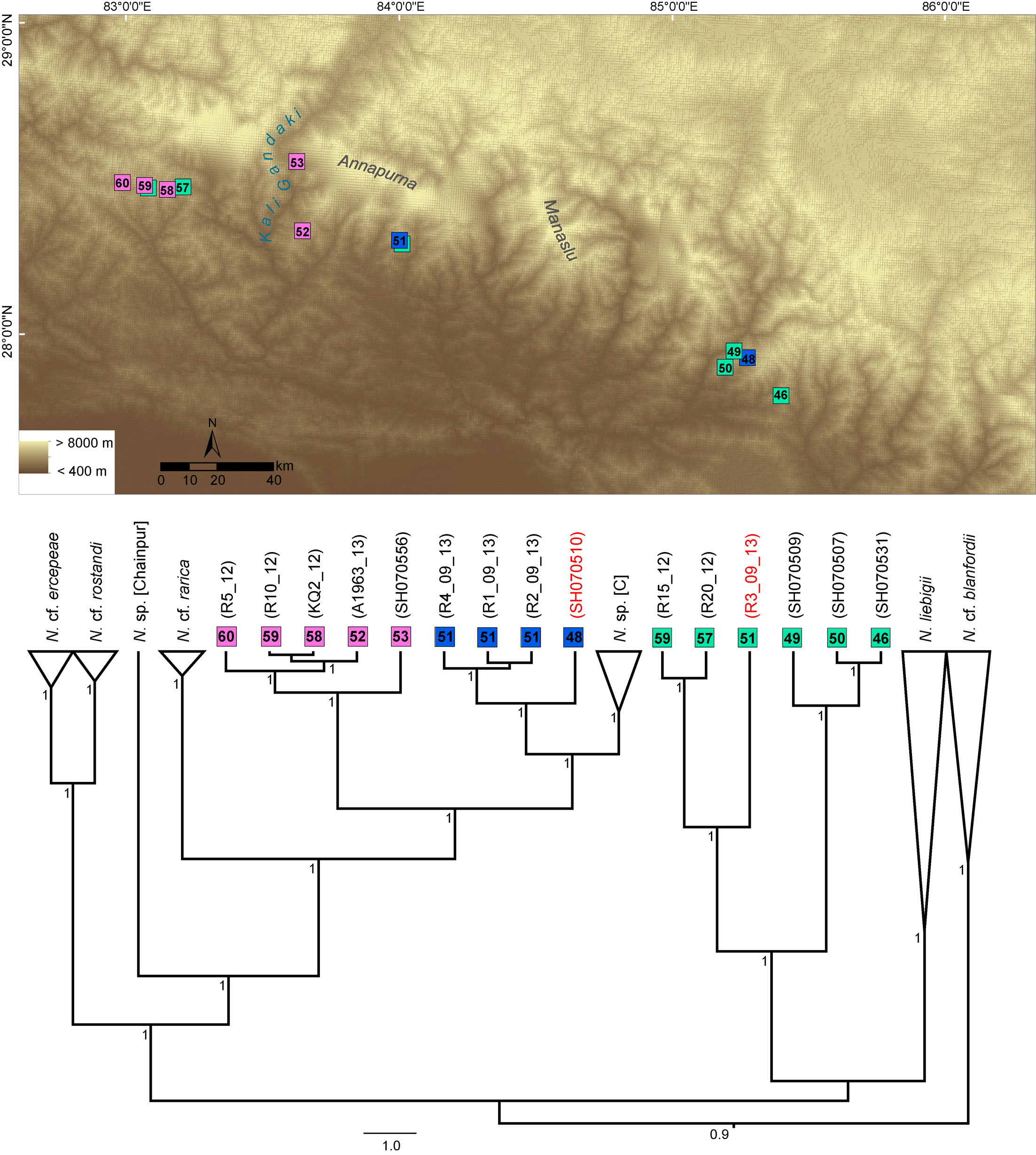

Supplement: Supplementary file 9 [file ECE3-9-14498-s009.tif]

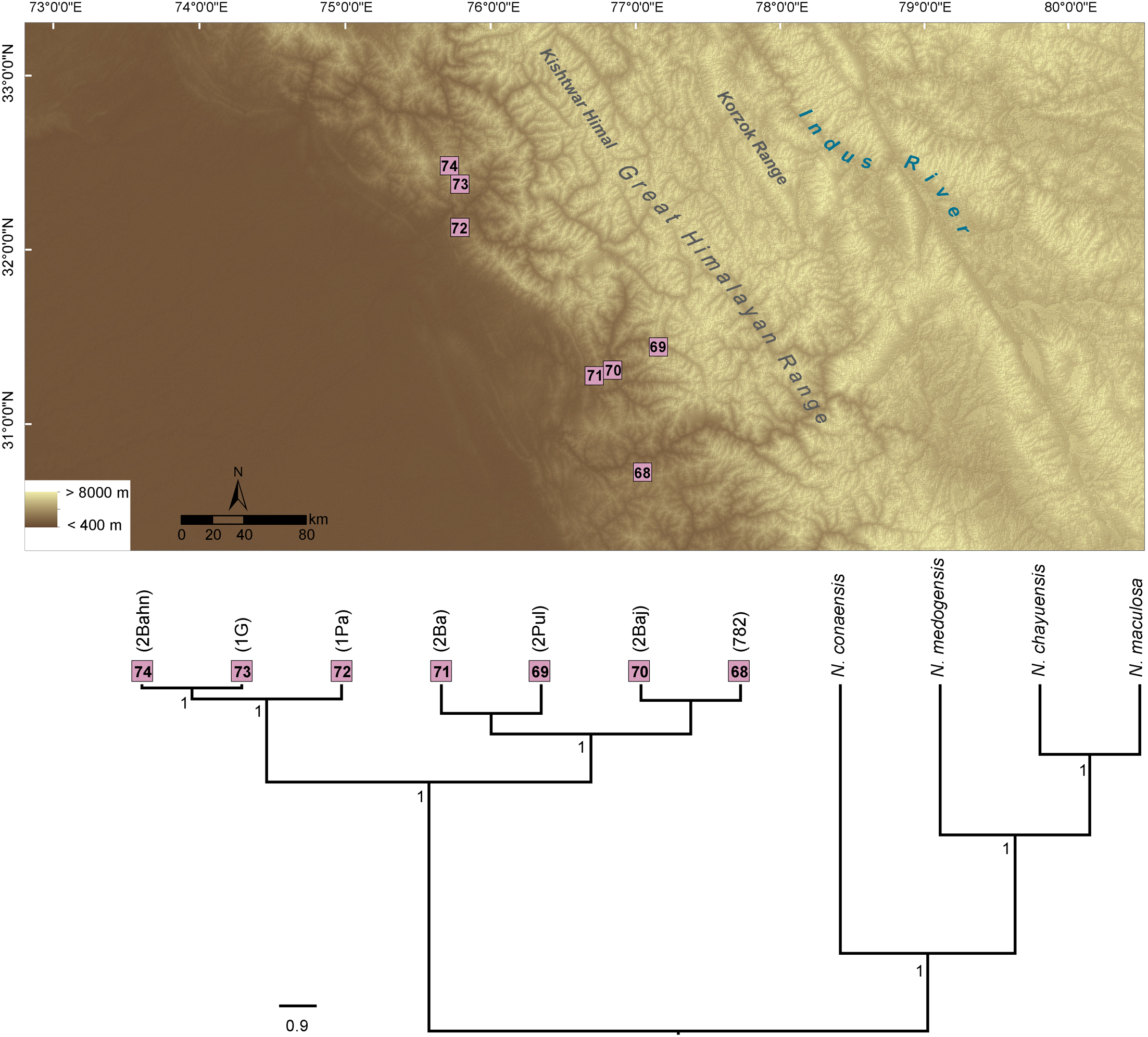

Supplement: Supplementary file 10 [file ECE3-9-14498-s010.tif]

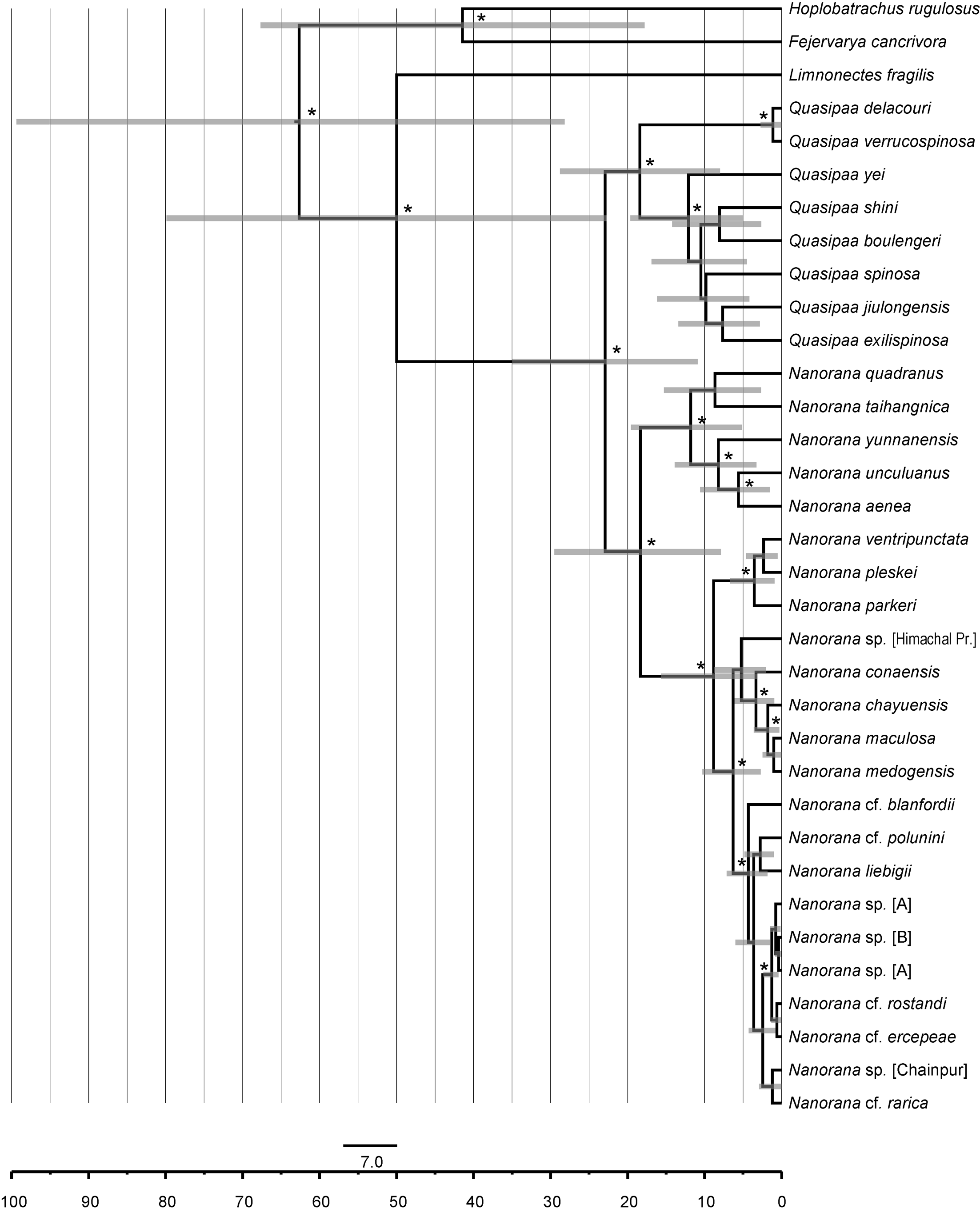

Supplement: Supplementary file 11 [file ECE3-9-14498-s011.tif]
