# Supplementary material for: Phylogeny of spiny frogs Nanorana (Anura: Dicroglossidae) supports a Tibetan origin of a Himalayan species group
Source: Ecol Evol. 2019 Dec 5;9(24):14498–511. doi: 10.1002/ece3.5909 (PMC6953589; doi:10.1002/ece3.5909)
Supplement: Supplementary file 2 [file ECE3-9-14498-s002.docx]

**APPENDIX 2**

**List of species, sample ID or voucher numbers, sample localities and GenBank accession numbers.** CAS = Chinese Academy of Science, NME = Natural History Museum of Erfurt, RAS = Russian Academy of Science. Locality numbers (no.) refer to the Fig. 1. Ancestral areas (AA) are defines as follows: A = NW Himalaya, B = Central Himalaya, C = East Himalaya; D = Transhimalaya and adjacent parts of the Tibetan Plateau; E = (sub)alpine parts of the eastern margin of the Tibetan Plateau; F = high montane regions of the south eastern margin of the Tibetan Plateau including the high mountains of Northern Vietnam; G = subtropical and meridional Eastern China; H = Sichuan Basin and mountains of Northeast China. Coordinates are given in decimal degrees.

| Taxon | AA | SampleID/voucher | Data origin | Locality no. | Lat | Long | 12S | 16S | co1 | rag1 | rhod | tyr |
| --- | --- | --- | --- | --- | --- | --- | --- | --- | --- | --- | --- | --- |
| *N. aenea* | F |  | NCBI | 3 | 22.336 | 103.844 | EU979693 | EU979830 | KR087830 | HM163609 | EU979895 | EU979986 |
| *N*. cf. *blanfordii* | B | JS040529_NME | this study | 29 | 27.617 | 87.233 | MN011966 | MN012067 | — | MN032491 | MN012330 | MN012480 |
| *N*. cf. *blanfordii* | B | JS040531_NME | this study | 29 | 27.617 | 87.233 | MN011967 | MN012068 | — | MN032492 | MN012331 | MN012481 |
| *N*. cf. *blanfordii* | B | JS040532_NME | this study | 29 | 27.617 | 87.233 | — | MN012069 | — | MN032493 | MN012332 | MN012482 |
| *N*. cf. *blanfordii* | B | JS040533_NME | this study | 29 | 27.617 | 87.233 | — | MN012070 | — | MN032494 | MN012333 | MN012483 |
| *N*. cf. *blanfordii* | B | JS040534_NME | this study | 29 | 27.617 | 87.233 | MN011968 | MN012071 |  | MN032495 | MN012334 | MN012484 |
| *N*. cf. *blanfordii* | B | JS040535_NME | this study | 29 | 27.617 | 87.233 | — | MN012072 | — | MN032496 | MN012335 | MN012485 |
| *N*. cf. *blanfordii* | B | JS060520_NME | this study | 28 | 27.173 | 87.421 | — | MN012073 | — | MN032497 | MN012336 | MN012486 |
| *N*. cf. *blanfordii* | B | JS060515_NME | this study | 27 | 27.214 | 87.463 | MN011970 | MN012074 | — | MN032498 | MN012337 | MN012487 |
| *N*. cf. *blanfordii* | B | JS060508_NME | this study | 25 | 27.413 | 87.734 | MN011969 | MN012075 | — | MN032499 | MN012338 | MN012488 |
| *N.* cf. *ercepeae* | B | A2016/13_NME | this study | 67 | 29.374 | 81.137 | MN011971 | — | MN012211 | — | MN012339 | MN012489 |
| *N.* cf. *ercepeae* | B | A2017/13_NME | this study | 67 | 29.374 | 81.137 | MN011972 | MN012076 | MN012212 | MN032500 | MN012340 | MN012490 |
| *N.* cf. *ercepeae* | B | A1_12_NME | this study | 63 | 28.963 | 82.857 | MN011973 | MN012077 | MN012213 | MN032501 | MN012341 | MN012491 |
| *N.* cf. *ercepeae* | B | A7_12_NME | this study | 62 | 28.855 | 82.961 | — | MN012078 | MN012214 | MN032502 | MN012342 | MN012492 |
| *N.* cf. *ercepeae* | B | A4_12_NME | this study | 61 | 28.857 | 82.976 | — | MN012079 | MN012215 | MN032503 | MN012343 | MN012493 |
| *N.* cf. *ercepeae* | B | A5_12_NME | this study | 61 | 28.857 | 82.976 | — | MN012080 | MN012216 | MN032504 | MN012344 | MN012494 |
| *N.* cf. *ercepeae* | B | A6_12_NME | this study | 61 | 28.857 | 82.976 | — | MN012081 | MN012217 | MN032505 | MN012345 | MN012495 |
| *N*. cf. *polunini* | B | R15_12_NME | this study | 59 | 28.502 | 83.129 | MN011974 | MN012082 | MN012218 | MN032506 | MN012346 | MN012496 |
| *N*. cf. *polunini* | B | R20_12_NME | this study | 57 | 28.513 | 83.255 | MN011975 | MN012083 | — | MN032507 | MN012347 | MN012497 |
| *N*. cf. *polunini* | B | SH070507_NME | this study | 50 | 28.060 | 85.294 | MN011976 | MN012084 | MN012219 | MN032508 | MN012348 | MN012498 |
| *N*. cf. *polunini* | B | SH070509_NME | this study | 49 | 28.080 | 85.295 | MN011977 | MN012085 | MN012220 | MN032509 | MN012349 | MN012499 |
| *N*. cf. *polunini* | B | SH070531_NME | this study | 46 | 27.965 | 85.472 | MN011978 | MN012086 | MN012221 | MN032510 | MN012350 | MN012500 |
| *N*. cf. *polunini* | B | R3_09_13_NME | this study | 51 | 28.380 | 84.065 | MN011979 | MN012087 | MN012222 | MN032511 | MN012351 | MN012501 |
| *N.* cf. *rarica* | B | A1961/13_NME | this study | 66 | 29.510 | 82.090 | MN012062 | MN012202 | MN012322 | — | MN012472 | MN012620 |
| *N.* cf. *rarica* | B | A1970/13_NME | this study | 66 | 29.510 | 82.090 | — | MN012203 | MN012323 | MN032606 | MN012473 | MN012621 |
| *N.* cf. *rarica* | B | A2015/13_NME | this study | 66 | 29.510 | 82.090 | — | MN012204 | MN012324 | MN032607 | MN012474 | MN012622 |
| *N.* cf. *rarica* | B | A2019/13_NME | this study | 66 | 29.510 | 82.090 | MN012063 | MN012205 | MN012325 | MN032608 | MN012475 | MN012623 |
| *N.* cf. *rarica* | B | A1965/13_NME | this study | 65 | 29.513 | 82.092 | MN012064 | MN012206 | MN012326 | MN032609 | MN012476 | MN012624 |
| *N.* cf. *rarica* | B | A1960/13_NME | this study | 64 | 29.360 | 82.200 | MN012065 | MN012207 | MN012327 | — | MN012477 | MN012625 |
| *N.* cf. *rarica* | B | A1961/13_NME | this study | 66 | 29.510 | 82.090 | MN012062 | MN012202 | MN012322 | — | MN012472 | MN012620 |
| *N.* cf. *rostandi* | B | R1_12_NME | this study | 60 | 28.513 | 83.033 | MN011980 | MN012088 | MN012223 | MN032512 | MN012352 | MN012502 |
| *N.* cf. *rostandi* | B | R2_12_NME | this study | 60 | 28.513 | 83.033 | MN011981 | MN012089 | MN012224 | MN032513 | MN012353 | MN012503 |
| *N.* cf. *rostandi* | B | R3_12_NME | this study | 60 | 28.513 | 83.033 | MN011982 | MN012090 | MN012225 | MN032514 | MN012354 | MN012504 |
| *N.* cf. *rostandi* | B | R4_12_NME | this study | 60 | 28.513 | 83.033 | — | MN012091 | MN012226 | MN032515 | MN012355 | MN012505 |
| *N.* cf. *rostandi* | B | R11_12_NME | this study | 59 | 28.502 | 83.129 | — | MN012092 | MN012227 | MN032516 | MN012356 | MN012506 |
| *N.* cf. *rostandi* | B | R12_12_NME | this study | 59 | 28.502 | 83.129 | — | MN012093 | MN012228 | MN032517 | MN012357 | MN012507 |
| *N.* cf. *rostandi* | B | R13_12_NME | this study | 59 | 28.502 | 83.129 | — | MN012094 | MN012229 | MN032518 | MN012358 | MN012508 |
| *N.* cf. *rostandi* | B | R14_12_NME | this study | 59 | 28.502 | 83.129 | — | MN012095 | MN012230 | MN032519 | MN012359 | MN012509 |
| *N.* cf. *rostandi* | B | R16_12_NME | this study | 59 | 28.502 | 83.129 | — | MN012096 | MN012231 | MN032520 | MN012360 | MN012510 |
| *N.* cf. *rostandi* | B | R6_12_NME | this study | 59 | 28.502 | 83.129 | — | MN012097 | MN012232 | MN032521 | MN012361 | MN012511 |
| *N.* cf. *rostandi* | B | R7_12_NME | this study | 59 | 28.502 | 83.129 | MN011983 | MN012098 | MN012233 | MN032522 | MN012362 | MN012512 |
| *N.* cf. *rostandi* | B | R8_12_NME | this study | 59 | 28.502 | 83.129 | MN011984 | MN012099 | MN012234 | MN032523 | MN012363 | MN012513 |
| *N.* cf. *rostandi* | B | R9_12_NME | this study | 59 | 28.502 | 83.129 | MN011985 | MN012100 | MN012235 | MN032524 | MN012364 | MN012514 |
| *N.* cf. *rostandi* | B | R17_12_NME | this study | 56 | 28.519 | 83.264 | MN011986 | MN012101 | MN012236 | MN032525 | MN012365 | MN012515 |
| *N.* cf. *rostandi* | B | SH070550_NME | this study | 55 | 28.683 | 83.591 | MN011987 | MN012102 | — | MN032526 | MN012366 | MN012516 |
| *N.* cf. *rostandi* | B | SH070538_NME | this study | 54 | 28.680 | 83.594 | MN011988 | MN012103 | — | MN032527 | MN012367 | MN012517 |
| *N. chayuensis* | F | SCUM050410CHX | NCBI | 12 | 25.823 | 98.858 | EU979714 | EU979838 | — | HM163587 | EU979854 | EU979945 |
| *N. conaensis* | C | KIZ-YP152 | NCBI | 16 | 27.991 | 91.957 | EU979703 | EU979834 | — | HM163589 | EU979874 | EU979965 |
| *N. liebigii* | B | A17_12_NME | this study | 56 | 28.519 | 83.264 | MN011989 | MN012104 | MN012237 | MN032528 | MN012368 | MN012518 |
| *N. liebigii* | B | R18_12_NME | this study | 56 | 28.519 | 83.264 | — | MN012105 | MN012238 | MN032529 | MN012369 | MN012519 |
| *N. liebigii* | B | SH070515_NME | this study | 47 | 28.099 | 85.317 | MN011990 | MN012106 | — | MN032530 | MN012370 | MN012520 |
| *N. liebigii* | B | SH0805109_NME | this study | 45 | 27.673 | 86.240 | MN011991 | MN012107 | — | MN032531 | MN012371 | MN012521 |
| *N. liebigii* | B | SH080506_NME | this study | 42 | 27.609 | 86.295 | MN011992 | MN012108 | — | MN032532 | MN012372 | — |
| *N. liebigii* | B | SH080554_NME | this study | 41 | 27.718 | 86.311 | MN011993 | MN012109 | — | MN032533 | MN012373 | MN012522 |
| *N. liebigii* | B | SH080536_NME | this study | 38 | 27.691 | 86.343 | — | MN012110 | MN012239 | — | MN012374 | MN012523 |
| *N. liebigii* | B | SH080537_NME | this study | 38 | 27.691 | 86.343 | MN011994 | MN012111 | MN012240 | MN032534 | MN012375 | MN012524 |
| *N. liebigii* | B | SH080538_NME | this study | 38 | 27.691 | 86.343 | MN011995 | MN012112 | — | MN032535 | MN012376 | MN012525 |
| *N. liebigii* | B | SH080524_NME | this study | 37 | 27.694 | 86.351 | MN011996 | MN012113 | MN012241 | MN032536 | MN012377 | MN012526 |
| *N. liebigii* | B | SH080534_NME | this study | 37 | 27.694 | 86.351 | MN011997 | MN012114 | — | MN032537 | MN012378 | MN012527 |
| *N. liebigii* | B | Ne16_13_NME | this study | 36 | 27.584 | 86.411 | MN011998 | MN012115 | MN012242 | MN032538 | MN012379 | MN012528 |
| *N. liebigii* | B | Ne17_13_NME | this study | 36 | 27.584 | 86.411 | — | MN012116 | MN012243 | MN032539 | MN012380 | MN012529 |
| *N. liebigii* | B | Ne12_13_NME | this study | 34 | 27.584 | 86.594 | MN011999 | MN012117 | MN012244 | MN032540 | MN012381 | MN012530 |
| *N. liebigii* | B | Ne10_13_NME | this study | 33 | 27.586 | 86.635 | MN012000 | MN012118 | MN012245 | MN032541 | MN012382 | MN012531 |
| *N. liebigii* | B | JS040512_NME | this study | 30 | 27.631 | 87.224 | MN012001 | MN012119 | MN012246 | MN032542 | MN012383 | MN012532 |
| *N. liebigii* | B | JS040513_NME | this study | 30 | 27.631 | 87.224 | MN012002 | MN012120 | MN012247 | MN032543 | MN012384 | MN012533 |
| *N. liebigii* | B | JS060518_NME | this study | 28 | 27.173 | 87.421 | — | MN012121 | — | MN032544 | MN012385 | MN012534 |
| *N. liebigii* | B | JS060511_NME | this study | 26 | 27.296 | 87.535 | MN012003 | MN012122 | — | MN032545 | MN012386 | MN012535 |
| *N. liebigii* | B | JS060509_NME | this study | 25 | 27.413 | 87.734 | — | MN012123 | — | MN032546 | MN012387 | MN012536 |
| *N. liebigii* | B | JS060502_NME | this study | 24 | 27.407 | 87.752 | MN012004 | — | — | MN032547 | MN012388 | MN012537 |
| *N. liebigii* | B | JS060503_NME | this study | 24 | 27.407 | 87.752 | MN012005 | MN012124 | — | MN032548 | MN012389 | MN012538 |
| *N. liebigii* | B | KIZ-RDXZL1 | NCBI | 23 | 27.485 | 88.907 | EU979702 | DQ118499 | KJ810987 | HM163607 | EU979864 | EU979955 |
| *N. maculosa* | F | YNU-HU2002308 | NCBI | 8 | 24.400 | 100.800 | EU979706 | EU979835 | — | HM163588 | EU979859 | EU979950 |
| *N. medogensis* | C | SYNU-XZ35 | NCBI | 13 | 29.367 | 95.583 | EU979705 | DQ118506 | — | HM163590 | EU979862 | EU979953 |
| *N. parkeri* | D | N6_06_NME | this study | 22 | 29.589 | 90.214 | — | MN012125 | MN012248 | — | MN012390 | MN012539 |
| *N. parkeri* | D | N7_06_NME | this study | 22 | 29.589 | 90.214 | MN012006 | MN012126 | MN012249 | MN032549 | MN012391 | MN012540 |
| *N. parkeri* | D | N8_06_NME | this study | 22 | 29.589 | 90.214 | — | MN012127 | MN012250 | MN032550 | MN012392 | MN012541 |
| *N. parkeri* | D | N5_06_NME | this study | 21 | 29.573 | 90.433 | MN012007 | MN012128 | MN012251 | — | MN012393 | MN012542 |
| *N. parkeri* | D | TP10_06_NME | this study | 21 | 29.573 | 90.433 | — | MN012129 | MN012252 | — | MN012394 | MN012543 |
| *N. parkeri* | D | TP11_06_NME | this study | 21 | 29.573 | 90.433 | MN012008 | MN012130 | MN012253 | — | MN012395 | MN012544 |
| *N. parkeri* | D | TP8_06_NME | this study | 21 | 29.573 | 90.433 | MN012009 | — | MN012254 | — | MN012396 | — |
| *N. parkeri* | D | TP9_06_NME | this study | 21 | 29.573 | 90.433 | MN012010 | MN012131 | MN012255 | — | MN012397 | MN012545 |
| *N. parkeri* | D | N10_06_NME | this study | 20 | 29.578 | 90.435 | MN012011 | MN012132 | MN012256 | — | MN012398 | MN012546 |
| *N. parkeri* | D | N9_06_NME | this study | 20 | 29.578 | 90.435 | — | MN012133 | MN012257 | MN032551 | MN012399 | MN012547 |
| *N. parkeri* | D | TP1_06_NME | this study | 20 | 29.578 | 90.435 | — | MN012134 | MN012258 | MN032552 | MN012400 | MN012548 |
| *N. parkeri* | D | TP2_06_NME | this study | 20 | 29.578 | 90.435 | — | MN012135 | — | MN032553 | MN012401 | MN012549 |
| *N. parkeri* | D | TP3_06_NME | this study | 20 | 29.578 | 90.435 | MN012012 | MN012136 | MN012259 | — | MN012402 | MN012550 |
| *N. parkeri* | D | CAS801L | this study | 19 | 30.090 | 90.480 | — | MN012137 | MN012260 | MN032554 | MN012403 | MN012551 |
| *N. parkeri* | D | CAS802L | this study | 19 | 30.090 | 90.480 | — | MN012138 | MN012261 | MN032555 | MN012404 | MN012552 |
| *N. parkeri* | D | CAS803L | this study | 19 | 30.090 | 90.480 | — | MN012139 | MN012262 | — | MN012405 | MN012553 |
| *N. parkeri* | D | CAS804L | this study | 19 | 30.090 | 90.480 | — | MN012140 | MN012263 | — | MN012406 | MN012554 |
| *N. parkeri* | D | CAS805L | this study | 19 | 30.090 | 90.480 | — | MN012141 | MN012264 | — | MN012407 | MN012555 |
| *N. parkeri* | D | A6AL_NME | this study | 18 | 30.156 | 90.647 | MN012013 | MN012142 | — | — | MN012408 | MN012556 |
| *N. parkeri* | D | JS0507B01_NME | this study | 17 | 30.378 | 90.908 | MN012014 | MN012143 | MN012265 | MN032556 | MN012409 | MN012557 |
| *N. parkeri* | D | JS0507B02_NME | this study | 17 | 30.378 | 90.908 | — | MN012144 | MN012266 | MN032557 | MN012410 | MN012558 |
| *N. parkeri* | D | JS0507B03_NME | this study | 17 | 30.378 | 90.908 | — | MN012145 | MN012267 | MN032558 | MN012411 | MN012559 |
| *N. parkeri* | D | JS0507B04_NME | this study | 17 | 30.378 | 90.908 | MN012015 | MN012146 | MN012268 | MN032559 | MN012412 | MN012560 |
| *N. parkeri* | D | JS0507B05_NME | this study | 17 | 30.378 | 90.908 | — | MN012147 | MN012269 | MN032560 | MN012413 | MN012561 |
| *N. parkeri* | D |  | NCBI | 17 | 30.378 | 90.908 | KP317482 | KP317482 | KP317482 | HM163584 | EU979873 | EU979964 |
| *N. parkeri* | D | N1_06_NME | this study | 15 | 31.166 | 92.061 | MN012016 | MN012148 | MN012270 | — | MN012414 | MN012562 |
| *N. parkeri* | D | N2_06_NME | this study | 15 | 31.166 | 92.061 | — | MN012149 | MN012271 | — | MN012415 | MN012563 |
| *N. parkeri* | D | N3_06_NME | this study | 15 | 31.166 | 92.061 | — | MN012150 | MN012272 | MN032561 | MN012416 | MN012564 |
| *N. parkeri* | D | N4_06_NME | this study | 15 | 31.166 | 92.061 | MN012017 | MN012151 | MN012273 | — | MN012417 | MN012565 |
| *N. parkeri* | D | TP4_06_NME | this study | 15 | 31.166 | 92.061 | — | MN012152 | MN012274 | — | MN012418 | MN012566 |
| *N. parkeri* | D | TP5_06_NME | this study | 15 | 31.166 | 92.061 | — | MN012153 | MN012275 | — | MN012419 | MN012567 |
| *N. parkeri* | D | TP6_06_NME | this study | 15 | 31.166 | 92.061 | MN012018 | MN012154 | MN012276 | — | MN012420 | MN012568 |
| *N. parkeri* | D | TP7_06_NME | this study | 15 | 31.166 | 92.061 | — | MN012155 | MN012277 | — | MN012421 | MN012569 |
| *N. parkeri* | D | CIB-XM1096 | NCBI | 14 | 29.649 | 94.361 | EU979722 | DQ118498 | KJ811345 | — | DQ458261 | DQ458276 |
| *N. pleskei* | E | KQ47_14_NME | this study | 6 | 30.216 | 101.500 | MN012019 | MN012156 | MN012278 | MN032562 | MN012422 | MN012570 |
| *N. pleskei* | E | KQ1_14_NME | this study | 5 | 30.377 | 101.675 | MN012020 | MN012157 | MN012279 | MN032563 | MN012423 | MN012571 |
| *N. pleskei* | E | KQ10_14_NME | this study | 5 | 30.377 | 101.675 | MN012021 | MN012158 | MN012280 | MN032564 | MN012424 | MN012572 |
| *N. pleskei* | E | KQ11_14_NME | this study | 5 | 30.377 | 101.675 | — | MN012159 | MN012281 | MN032565 | MN012425 | MN012573 |
| *N. pleskei* | E | KQ13_14_NME | this study | 5 | 30.377 | 101.675 | MN012022 | MN012160 | MN012282 | MN032566 | MN012426 | MN012574 |
| *N. pleskei* | E | KQ15_14_NME | this study | 5 | 30.377 | 101.675 | — | MN012161 | MN012283 | — | MN012427 | MN012575 |
| *N. pleskei* | E | KQ17_14_NME | this study | 5 | 30.377 | 101.675 | MN012023 | MN012162 | MN012284 | MN032567 | MN012428 | MN012576 |
| *N. pleskei* | E | KQ18_14_NME | this study | 5 | 30.377 | 101.675 | — | MN012163 | MN012285 | MN032568 | MN012429 | MN012577 |
| *N. pleskei* | E | KQ19_14_NME | this study | 5 | 30.377 | 101.675 | — | MN012164 | MN012286 | — | MN012430 | MN012578 |
| *N. pleskei* | E | KQ20_14_NME | this study | 5 | 30.377 | 101.675 | — | MN012165 | MN012287 | MN032569 | MN012431 | MN012579 |
| *N. pleskei* | E | KQ9_14_NME | this study | 5 | 30.377 | 101.675 | MN012024 | MN012166 | MN012288 | — | MN012432 | MN012580 |
| *N. pleskei* | E | CAS201 | this study | 4 | 33.467 | 102.750 | MN012025 | MN012167 | MN012289 | MN032570 | MN012433 | MN012581 |
| *N. pleskei* | E | CAS202 | this study | 4 | 33.467 | 102.750 | MN012026 | MN012168 | MN012290 | MN032571 | MN012434 | MN012582 |
| *N. pleskei* | E |  | NCBI | 4 | 33.467 | 102.750 | HQ324232 | HQ324232 | HQ324232 | HM163586 | EU979870 | EU979961 |
| *N. quadranus* | H | SCUM20045195CJ | NCBI | 2 | 31.683 | 103.850 | EU979695 | DQ118514 | — | HM163591 | EU979887 | EU979978 |
| *N.* sp. [A] | B | R5_12_NME | this study | 60 | 28.513 | 83.033 | MN012027 | MN012169 | MN012291 | MN032572 | MN012435 | MN012583 |
| *N.* sp. [A] | B | R10_12_NME | this study | 59 | 28.502 | 83.129 | MN012028 | MN012170 | — | MN032573 | MN012436 | MN012584 |
| *N.* sp. [A] | B | KQ2_12_NME | this study | 58 | 28.501 | 83.198 | MN012029 | MN012171 | MN012292 | MN032574 | MN012437 | MN012585 |
| *N.* sp. [A] | B | SH070556_NME | this study | 53 | 28.622 | 83.662 | MN012030 | MN012172 | MN012293 | MN032575 | MN012438 | MN012586 |
| *N.* sp. [A] | B | A1963/13_NME | this study | 52 | 28.400 | 83.700 | MN012031 | MN012173 | MN012294 | — | MN012439 | MN012587 |
| *N.* sp. [B] | B | R1_09_13_NME | this study | 51 | 28.380 | 84.065 | MN012032 | MN012174 | MN012295 | MN032576 | MN012440 | MN012588 |
| *N.* sp. [B] | B | R2_09_13_NME | this study | 51 | 28.380 | 84.065 | MN012033 | MN012175 | MN012296 | MN032577 | MN012441 | MN012589 |
| *N.* sp. [B] | B | R4_09_13_NME | this study | 51 | 28.380 | 84.065 | MN012034 | MN012176 | MN012297 | MN032578 | MN012442 | MN012590 |
| *N.* sp. [B] | B | SH070510_NME | this study | 48 | 28.074 | 85.302 | MN012035 | MN012177 | — | MN032579 | MN012443 | MN012591 |
| *N.* sp. [C] | B | SH080591_NME | this study | 44 | 27.686 | 86.252 | MN012036 | MN012178 | MN012298 | MN032580 | MN012444 | MN012592 |
| *N.* sp. [C] | B | SH080592_NME | this study | 44 | 27.686 | 86.252 | MN012037 | MN012179 | MN012299 | MN032581 | MN012445 | MN012593 |
| *N.* sp. [C] | B | SH080593_NME | this study | 44 | 27.686 | 86.252 | MN012038 | MN012180 | MN012300 | MN032582 | MN012446 | MN012594 |
| *N.* sp. [C] | B | SH080594_NME | this study | 44 | 27.686 | 86.252 | MN012039 | MN012181 | MN012301 | MN032583 | MN012447 | MN012595 |
| *N.* sp. [C] | B | SH080570_NME | this study | 43 | 27.697 | 86.275 | MN012040 | MN012182 | MN012302 | MN032584 | MN012448 | MN012596 |
| *N.* sp. [C] | B | SH080571_NME | this study | 43 | 27.697 | 86.275 | MN012041 | MN012183 | MN012303 | MN032585 | MN012449 | MN012597 |
| *N.* sp. [C] | B | SH080572_NME | this study | 43 | 27.697 | 86.275 | MN012042 | MN012184 | MN012304 | MN032586 | MN012450 | MN012598 |
| *N.* sp. [C] | B | SH080553_NME | this study | 41 | 27.718 | 86.311 | MN012043 | MN012185 | MN012305 | MN032587 | MN012451 | MN012599 |
| *N.* sp. [C] | B | SH080555_NME | this study | 41 | 27.718 | 86.311 | MN012044 | MN012186 | MN012306 | MN032588 | MN012452 | MN012600 |
| *N.* sp. [C] | B | SH080545_NME | this study | 40 | 27.703 | 86.337 | MN012045 | MN012187 | MN012307 | MN032589 | MN012453 | MN012601 |
| *N.* sp. [C] | B | SH080546_NME | this study | 40 | 27.703 | 86.337 | MN012046 | MN012188 | MN012308 | MN032590 | MN012454 | MN012602 |
| *N.* sp. [C] | B | SH080548_NME | this study | 40 | 27.703 | 86.337 | MN012047 | MN012189 | MN012309 | MN032591 | MN012455 | MN012603 |
| *N.* sp. [C] | B | SH080551_NME | this study | 40 | 27.703 | 86.337 | MN012048 | MN012190 | MN012310 | MN032592 | MN012456 | MN012604 |
| *N.* sp. [C] | B | SH080552_NME | this study | 40 | 27.703 | 86.337 | MN012049 | MN012191 | MN012311 | — | MN012457 | MN012605 |
| *N.* sp. [C] | B | SH080512_NME | this study | 39 | 27.595 | 86.340 | MN012050 | MN012192 | MN012312 | MN032593 | MN012458 | MN012606 |
| *N.* sp. [C] | B | SH080523_NME | this study | 37 | 27.694 | 86.351 | MN012051 | MN012193 | MN012313 | MN032594 | MN012459 | MN012607 |
| *N.* sp. [C] | B | Ne13_13_NME | this study | 35 | 27.576 | 86.514 | MN012052 | MN012194 | MN012314 | MN032595 | MN012460 | MN012608 |
| *N.* sp. [C] | B | Ne1_13_NME | this study | 32 | 27.689 | 86.731 | MN012053 | MN012195 | MN012315 | MN032596 | MN012461 | MN012609 |
| *N.* sp. [C] | B | Ne2_13_NME | this study | 32 | 27.689 | 86.731 | — | MN012196 | MN012316 | MN032597 | MN012462 | MN012610 |
| *N.* sp. [C] | B | Ne9_13_NME | this study | 31 | 27.671 | 86.765 | MN012054 | MN012197 | MN012317 | MN032598 | MN012463 | MN012611 |
| *N.* sp. [Chainpur Himal] | B | A1966/13_NME | this study | 67 | 29.374 | 81.137 | MN012055 | MN012198 | MN012318 | MN032599 | MN012464 | MN012612 |
| *N.* sp. [Himachal Pradesh] | A | 2Bhan_RAS | this study | 74 | 32.873 | 75.858 | MN012056 | MN012199 | MN012319 | — | MN012465 | MN012613 |
| *N.* sp. [Himachal Pradesh] | A | 1G_RAS | this study | 73 | 32.777 | 75.947 | MN012057 | — | MN012320 | MN032600 | MN012466 | MN012614 |
| *N.* sp. [Himachal Pradesh] | A | 1Pa_RAS | this study | 72 | 32.528 | 75.991 | MN012058 | — | MN012321 | MN032601 | MN012467 | MN012615 |
| *N.* sp. [Himachal Pradesh] | A | 2Ba_RAS | this study | 71 | 31.783 | 77.068 | MN012059 | — | — | MN032602 | MN012468 | MN012616 |
| *N.* sp. [Himachal Pradesh] | A | 2Baj_RAS | this study | 70 | 31.821 | 77.112 | — | — | — | MN032603 | MN012469 | MN012617 |
| *N.* sp. [Himachal Pradesh] | A | 2Pul_RAS | this study | 69 | 31.996 | 77.448 | MN012060 | MN012200 | — | MN032604 | MN012470 | MN012618 |
| *N.* sp. [Himachal Pradesh] | A | 782_RAS | this study | 68 | 31.261 | 77.450 | MN012061 | MN012201 | — | MN032605 | MN012471 | MN012619 |
| *N. taihangnica* | H |  | NCBI | 1 | 35.265 | 112.090 | KF199146 | KF199146 | KF199146 | HM163608 | EU979893 | EU979984 |
| *N. unculuanus* | F | YNUHU2002502601 | NCBI | 7 | 24.447 | 100.834 | EU979699 | DQ118491 | — | HM163595 | DQ458262 | DQ458277 |
| *N. ventripunctata* | E | SCUM045887WD | NCBI | 11 | 27.830 | 99.701 | EU979717 | EU979839 | KJ810985 | HM163585 | EU979868 | EU979959 |
| *N. ventripunctata* | E | SH050538_NME | this study | 10 | 27.788 | 99.855 | MN012066 | MN012208 | MN012328 | MN032610 | MN012478 | MN012626 |
| *N. ventripunctata* | E | SH050539_NME | this study | 10 | 27.788 | 99.855 | — | MN012209 | MN012329 | MN032611 | MN012479 | MN012627 |
| *N. yunnanensis* | F |  | NCBI | 9 | 27.724 | 100.789 | KF199150 | KF199150 | KF199150 | HM163593 | EU979875 | EU979966 |
| *Q. boulengeri* | G | YNU-HU20025106 | NCBI | 80 | 28.811 | 105.831 | KX645665 | KX645665 | KX645665 | HM163604 | EU979914 | EU980005 |
| *Q. delacouri* | G | FMNH255623 | NCBI | 82 | 19.018 | 104.799 | — | EU979810 | EU979664 | HM163600 | EU979900 | EU979991 |
| *Q. exilispinosa* | G | KF199151 | NCBI | 81 | 22.396 | 114.109 | KF199151 | KF199151 | KF199151 | HM163610 | EU979922 | EU980013 |
| *Q. jiulongensis* | G | KF199149 | NCBI | 75 | 27.750 | 117.683 | KF199149 | KF199149 | KF199149 | HM163603 | EU979927 | EU980018 |
| *Q. shini* | G | KF199148 | NCBI | 76 | 25.598 | 109.935 | KF199148 | KF199148 | KF199148 | HM163602 | EU979907 | EU979998 |
| *Q. spinosa* | F |  | NCBI | 77 | 24.481 | 99.047 | NC_013270 | NC_013270 | NC_013270 | HM163606 | EU979913 | EU980004 |
| *Q. verrucospinosa* | G |  | NCBI | 78 | 21.789 | 101.142 | KF199147 | KF199147 | KF199147 | HM163599 | EU979896 | EU979987 |
| *Q. yei* | G | HNNU0908I061 | NCBI | 79 | 31.798 | 115.407 | KJ842105 | KJ842105 | KJ842105 | HM163596 | EU979905 | EU979996 |
| *Fejervarya cancrivora* | G |  | NCBI |  |  |  | EU652694 | EU652694 | EU652694 | HM163581 | EU979938 | EU980029 |
| *Hoplobatrachus rugulosus* | G |  | NCBI |  |  |  | NC_019615 | NC_019615 | NC_019615 | HM163612 | EU979933 | EU980024 |
| *Limnonectes fragilis* | G | ZNAC11006 | NCBI |  |  |  | AY899241 | AY899241 | AY899241 | HM163611 | DQ458270 | DQ458285 |
| *Phrynobatrachus natalensis* |  |  | NCBI |  |  |  | DQ283414 | KY177049 | — | KX208767 | DQ284019 | — |
| *Ptychadena oxyrhynchus* |  |  | NCBI |  |  |  | AF215205 | KY177055 | DQ525954 | KX208773 | — | — |
| *Sooglossus thomasseti* |  |  | NCBI |  |  |  | JX564895 | JX564895 | JF703233 | AY323778 | AY323744 | AY341761 |
| *Dryophytes cinereus* |  |  | NCBI |  |  |  | AY819366 | AY330892 | KU985703 | AY323766 | AY323749 | AY844063 |
| *Hyla annectans* |  |  | NCBI |  |  |  | KM271781 | KM271781 | KM271781 | AY844388 | AY844574 | AY844045 |
| *Kaloula pulchra* |  |  | NCBI |  |  |  | — | MG935853 | MG935559 | EF396091 | DQ284011 | EF395978 |
| *Microhyla heymonsi* |  |  | NCBI |  |  |  | — | MG935912 | MG935618 | EF396095 | — | EF395979 |
