# Supplementary material for: Phylogeny of spiny frogs Nanorana (Anura: Dicroglossidae) supports a Tibetan origin of a Himalayan species group
Source: Ecol Evol. 2019 Dec 5;9(24):14498–511. doi: 10.1002/ece3.5909 (PMC6953589; doi:10.1002/ece3.5909)
Supplement: Supplementary file 3 [file ECE3-9-14498-s003.docx]

**APPENDIX 3**

**Table S1.2.** **Primer and annealing temperature used for DNA amplification (amp) and sequencing (seq).**

| *gene* | *Primer* |  | *Direction* | *Sequence 5’ → 3’* | *Annealing [°C]* | *Reference* |
| --- | --- | --- | --- | --- | --- | --- |
| *12S ribosomal RNA* (*12S*) | FS01 | amp + seq | forward | AACGCTAAGATGAACCCTAAAAAGTTCT | 55.0 | 1 |
|  | R16 | amp + seq | reverse | ATAGTGGGGTATCTAATCCCAGTTTGTTTT | 55.0 | 1 |
| *16S ribosomal RNA* (*16S*) | 16Sar | amp + seq | forward | CGCCTGTTTATCAAAAACAT | 51.1 | 2 |
|  | 16Sbr | amp + seq | reverse | CCGGTCTGAACTCAGATCACGT | 62.1 | 2 |
|  | HO3063 | amp + seq | reverse | CTCCGGTTTGAACTCAGATC | 57.3 | 3 |
| *cytochrome oxidase subunit 1* (*co1*) | Chmf4 | amp + seq | forward | TYTCWACWAAYCAYAAAGAYATCGG | 58.1 | this study |
|  | Chmr4 | amp + seq | reverse | ACYTCRGGRTGRCCRAARAATCA | 60.6 | this study |
| *recombination activating protein 1 gene* (*rag1*) | Rag1N-01f | amp + seq | forward | GGGTATCATCCCTTTGAATGGAAAC | 61.3 | this study |
|  | Rag1N-01r | amp + seq | reverse | CCAATGGAACCATCGCGTTC | 59.4 | this study |
| *rhodopsin (rhod)* | Rhod1A | amp + seq | forward | ACCATGAACGGAACAGAAGGYCC | 63.3 | 4 |
|  | Rhod1D | amp + seq | reverse | GTAGCGAAGAARCCTTCAAMGTA | 58.9 | 4 |
| *tyrosinase (tyr)* | TyrC | amp + seq | forward | GGCAGAGGAWCRTGCCAAGATGT | 63.3 | 4 |
|  | TyrG | amp + seq | reverse | TGCTGGGCRTCTCTCCARTCCCA | 66.0 | 4 |

**References**

1. Sumida et al. (1998) Evolutionary relationships among Japanese pond frogs inferred from mitochondrial DNA sequences of cytochrome b and 12S ribosomal RNA genes. Genes and Genetic Systems 73:121–133.
2. Palumbi (1996). Nucleic acids II: the polymerase chain reaction. In: Hillis, Moritz et Mable (Eds), Molecular systematics. Sinauer Associates, Sunderland, pp. 205–247.
3. Xiong et Kocher (1991) Comparison of mitochondrial DNA sequences of seven morphospecies of black flies (Diptera: Simuliidae). Genome 34(2):306–11.
4. Bossuyt et. Milinkowich (2000) Convergent adaptive radiations in Madagascan and Asian ranid frogs reveal covariation between larval and adult traits. PNAS 97(12):6585–6590.
