# Supplementary material for: Phylogeny of spiny frogs Nanorana (Anura: Dicroglossidae) supports a Tibetan origin of a Himalayan species group
Source: Ecol Evol. 2019 Dec 5;9(24):14498–511. doi: 10.1002/ece3.5909 (PMC6953589; doi:10.1002/ece3.5909)
Supplement: Supplementary file 6 [file ECE3-9-14498-s006.docx]

**APPENDIX 5**

**Summarized results of the topological tests of the two different topologies for the concatenated data set.** Marginal likelihoods for the Bayes Factor (BF) model selection approach were estimated based on the stepping stone (ss) and path sampling (ps) method in BEAST v.1.10.4; using both the optimale scheme based on **(a)** gene fragments and **(b)** gene+codon positions for protein-coding genes. CH = Central Himalaya clade; EH = East Himalaya Clade; NWH = Northwestern Himalaya clade. Best-supported model is marked bold. A 2lnBF = 0–2 means “not worth more than a bare mention”, 2lnBf = 2–6 means “positive” support, 2lnBf = 6–10 provides “strong” support, and 2lnBf >10 means “decisive” support.

|  | *Partition scheme genes* | | *Partition scheme genes + codons* | |
| --- | --- | --- | --- | --- |
| *Model* | *ps* | *ss* | *ps* | *ss* |
| **m1 ((NWH(CH))ingroup)** | -22392.7 | -22396.2 | -22048.0 | -22056.0 |
| m2 ((NWH(EH))ingroup) | -22484.3 | -22488.1 | -22121.9 | -22121.0 |
| 2lnBF m1:m2 | 183.2 | 183.8 | 147.8 | 130.0 |
