# Supplementary material for: Phylogeny of spiny frogs Nanorana (Anura: Dicroglossidae) supports a Tibetan origin of a Himalayan species group
Source: Ecol Evol. 2019 Dec 5;9(24):14498–511. doi: 10.1002/ece3.5909 (PMC6953589; doi:10.1002/ece3.5909)
Supplement: Supplementary file 12 [file ECE3-9-14498-s012.docx]

**APPENDIX 8**

**Age estimates (in million years) for selected nodes referenced in Figure 4 and S2.** CSD = concatenated sequence analysis; MSC = multispecies coalescent model. Subgenus *Chaparana* = *Nanorana* species east of the Himalaya-Tibet-Orogen; Subgenus *Nanorana* = *Nanorana* species at the Tibetan Plateau and eastern slope of it; Subgenus *Paa* = *Nanorana* species in East Tibet and the Greater Himalaya.

|  | ***Roelants et al. 2004*** [CDS] | ***Bossuyt et al. 2006*** [CDS] | ***Wiens et al. 2009***  [CDS] | ***Che et al. 2010*** [CDS] | ***Chen et al. 2017*** [CDS] | ***Sun et al. 2018*** [CDS) | ***this study*** [MSC] |
| --- | --- | --- | --- | --- | --- | --- | --- |
|  | mt: 12S, tRNA^Val^, 16S; nu: rhod, tyr | nu: rag1, rhod, tyr | mt: 12S, tRNA^Val^, 16S; nu: rag1, rhod, tyr | nu: rag1, rag2, rhod, tyr | mt genome* | transcriptome | nu: rag1, rhod, tyr |
| *Hoplobatrachus vs. Feijervarya* | 40 | 39 | 49 | 46 | 38 |  | 41.4 (17.8-67.7) |
| *MRCA Hoplobatrachus/(Limnonectes)/*  *Feijervarya/Quasipaa* | 60 | 63 (67) | 62 (54) | 70 | 53 (44) |  | 62.7 (28.2-99.4) |
| *MRCA* *Quasipaa* |  |  |  | 24 |  |  | 18.4 (8.0-28.7) |
| *MRCA Quasipaa and Nanorana* | 41 | 38.1 | 29 | 27 | 30 | 22.4 (11.5-33.8) | 22.9 (10.9-35.0) |
| *Chaparana vs. Paa+Nanorana* | 25 | 30 | 10-18 | 23 | 15 |  | 18.3 (7.9-29.5) |
| *Nanorana vs. Paa* |  |  | 10-12 | 19 |  | 13.1 (6.9-24.6) | 8.8 (3.2-15.6) |
| *Nanorana parkeri vs. N. pleskei* | 12 |  | 6 | 9 | 8 |  | 3.5 (0.9-6.7) |

*13 protein-coding genes without all third codon positions, two rRNAs, and the concatenated 14 tRNAs

**References**

Bossuyt et al. (2006) Phylogeny and Biogeography of a Cosmopolitan Frog Radiation: Late Cretaceous Diversification Resulted in Continent-Scale Endemism in the Family Ranidae. Systematic Biology 55(4):579-594.

Che et al. (2010) Spiny frogs (Paini) illuminate the history of the Himalayan region and Southeast Asia. Proc Natl Acad Sci USA 107(31):13765-13770.

Chen et al. (2017) Molecular phylogeny of the family Dicroglossidae (Amphibia: Anura) inferred from complete mitochondrial genomes. Biochemical Systematics and Ecology 71:1-9.

Roelants et al. (2004) Endemic ranid (Amphibia: Anura) genera in southern mountain ranges of the Indian subcontinent represent ancient frog lineages: evidence from molecular data. Molecular Phylogenetics and Evolution 31:730-740.

Sun et al. (2018) Species groups distributed across elevational gradients reveal convergent and continuous genetic adaptation to high elevations. Proc Natl Acad Sci USA 115(45):E10634-E10641.

Wiens et al. (2009) Evolutionary and biogeographic origins of high tropical diversity in Old World frogs (Ranidae). Evolution 63(5):1217-31.
