# Supplementary material for: Phylogeny of spiny frogs Nanorana (Anura: Dicroglossidae) supports a Tibetan origin of a Himalayan species group
Source: Ecol Evol. 2019 Dec 5;9(24):14498–511. doi: 10.1002/ece3.5909 (PMC6953589; doi:10.1002/ece3.5909)
Supplement: Supplementary file 13 [file ECE3-9-14498-s013.docx]

**APPENDIX 1**

**Map of High Asia.** Main parts of the Himalayan Tibetan mountain system and known records of the 30 accepted *Nanorana* species (Frost, 2019; sources: Che et al., 2009; Che et al., 2010; Grosjean et al., 2015; Huang et al., 2016; Rais et al., 2014; Rizvi et al., 2012; Wang et al., 2009; Wang et al., 2012; Yang et al., 2011; Zhang et al., 2010; Zhou et al., 2014; vertnet.org; ASW 6.0 [Frost, 2019]). Our own records of *N. liebigii*, *N. parkeri*, *N. pleskei*, and *N. ventripunctata* are also included in the map, of which the 16S sequences matched NCBI entries of the respective species to at least 99% (we don’t distinguish them in the figure to avoid making the colour code too complex). Subgenera as yielded from our analyses: *Chaparana: N. aenea, N. quadranus, N. taihangnica, N. unculuanus, N. ­­yunnanensis*; *Nanorana*: *N. parkeri, N. pleskei, N. ventripunctata*; *Paa*: *N.* (cf.) *blanfordii*, *N.* *chayuensis, N.* *conaensis, N.* (cf.) *ercepeae, N.* *liebigii, N.* *maculosa, N.* *medogensis, N.* (cf.) *polunini, N.* (c.f.) *rarica, N.* (cf.) *rostandi*.

**References Appendix 1**

Che, J., Hu, J.S., Zhou, W.W., Murphy, R.W., Papenfuss, T.J., Chen, M.Y., Rao, D.Q., Li, P.P., Zhang, Y.P., 2009. Phylogeny of the Asian spiny frog tribe Paini (Family Dicroglossidae) sensu Dubois. Molecular Phylogenetics and Evolution 50, 59–73.

Che, J., Zhou, W.W., Hu, J.S., Yan, F., Papenfuss, T.J., Wake, D.B., Zhang, Y.P., 2010. Spiny frogs (Paini) illuminate the history of the Himalayan region and Southeast Asia. Proceedings of the National Academy of Sciences of the United States of America 107, 13765–13770.

Frost, D.R., 2019. Amphibian species of the world: an online reference. Version 6.0. Electronic Database accessible at http://research.amnh.org/herpetology/amphibia/index.html. American Museum of Natural History, New York, USA.

Grosjean, S., Ohler, A., Chuaynkern, Y., Cruaud, C., Hassanin, A., 2015. Improving biodiversity assessment of anuran amphibians using DNA barcoding of tadpoles. Case studies from Southeast Asia. Comptes Rendus Biologies 338, 708.

Huang, Y., Hu, J., Wang, B., Song, Z., ZHOU, Z.Y., Jiang, J., 2016. Integrative taxonomy helps to reveal the mask of the genus *Gynandropaa* (Amphibia: Anura: Dicroglossidae) Integrative Zoology 11, 134–150.

Rais, M., Abbassi, S., Batool, T., Jilani, M.J., Assadi, M.A., Mubarak, H., Baloch, S., 2014. A note on recapture of *Nanorana vicina* (Anura: Amphibia) from Murree, Pakistan. The Journal of Animal & Plant Sciences 24, 455–458.

Rizvi, A.N., Bursey, C.R., Bhutia, P.T., 2012. Three new species of Digenea (Batrachotrematidae) in *Nanorana minica* (Anura, Dicroglossidae) from Uttarakhand, India. Acta Parasitologica 57, 154–159.

Wang, B., Jiang, J., Xie, F., Chen, X., Dubois, A., Liang, G., Wagner, S., 2009. Molecular phylogeny and genetic identification of populations of two Species of *Feirana* frogs (Amphibia: Anura, Ranidae, Dicroglossinae, Paini) Endemic to China. Zool Sci 26, 500–509.

Wang, B., Jiang, J., Xie, F., Li, C., 2012. Postglacial colonization of the Qinling Mountains: phylogeography of the swelled vent frog (*Feirana quadranus*). PLoS One 7, e41579.

Yang, X., Wang, B., Hu, J., Jiang, J., 2011. A new species of the genus *Feirana* (Amphibia: Anura: Dicroglossidae) from the western Qinling Mountains of China. Asian Herpetological Research 2011/2, 72–86

Zhang, D.R., Chen, M.Y., Murphy, R.W., Che, J., Pang, J.F., Hu, J.S., Luo, J., Wu, S.J., Ye, H., Zhang, Y.P., 2010. Genealogy and palaeodrainage basins in Yunnan Province: phylogeography of the Yunnan spiny frog, *Nanorana yunnanensis* (Dicroglossidae). Molecular Ecology 19, 3406–3420.

Zhou, W.W., Zhang, B.L., Chen, H.M., Jin, J.Q., Yang, J.X., Wang, Y.Y., Jiang, K., Murphy, R.W., Zhang, Y.P., Che, J., 2014. DNA barcodes and species distribution models evaluate threats of global climate changes to genetic diversity: a case study from *Nanorana parkeri* (Anura: Dicroglossidae). PLoS One 9, e103899.

**APPENDIX 4**

**Clades identified by network analysis based (a) on the concatenated mitochondrial and nuclear sequence DNA and (b) on the nuclear data only.** Colour code for Himalayan samples (darker grey shaded area in [a]) corresponds to Figures 1 and S1.1 in Appendix 1 (for details see text). Species of the Tibetan Plateau (*N. parkeri*, *N. pleskei*, *N. ventripunctata*) are indicated in (a) by the lighter grey shaded area.

**APPENDIX 6**

**Comparison of phylogeny (based in mtDNA+nuDNA) and geographical distribution of *Nanorana* lineages. (A)** *Nanorana liebigii*, **(B)** *N.* cf. *blanfordii* (brown) and *N.* sp. [C] (grey-blue), and **(C)** *N.* sp. [A] (pink), *N.* sp. [B] (royal) and *N.* cf. *polunini* (cyan) in the Central Himalaya as well as **(D)** *N.* sp. in the NW Himalaya. Below each map: Bayesian inference tree based on concatenated mtDNA and nuDNA sequence data (for the tree incl. outgroups see Fig. 2 in the main text). Numbers on branch nodes are posterior probability values ≥ 0.80. Specifications in brackets refer to the internal specimen identification code. Specimens marked in red letters suggest more recent dispersal events, probably along river gorges. The sampling localities of haplotypes are shown in the map above. Haplotypes belonging to the same lineage are indicated by coloured boxes.

**APPENDIX 7**

**Time calibrated species tree of Asian spiny frogs using *BEAST and based on nuDNA.** Bayesian posterior probability values ≥ 95% are indicated by a star at the respective node. Codes in square brackets next to undescribed lineages (*N.* sp.) specifies their region of origin (HimPr = Himachal Pradesh; Chainpur = Chainpur Himal; Rara Lake) or simply a working label (A, B, C). Taxa for fossil calibration scheme were excluded for readability.
